# A Complex Systems Approach to Causal Discovery in Psychiatry

**DOI:** 10.1371/journal.pone.0151174

**Published:** 2016-03-30

**Authors:** Glenn N. Saxe, Alexander Statnikov, David Fenyo, Jiwen Ren, Zhiguo Li, Meera Prasad, Dennis Wall, Nora Bergman, Ernestine C. Briggs, Constantin Aliferis

**Affiliations:** 1 Department of Child and Adolescent Psychiatry, New York University School of Medicine, New York, New York, United States of America; 2 Center for Health Informatics and Bioinformatics, New York University School of Medicine, New York, New York, United States of America; 3 Memorial Sloan Kettering Cancer Center, New York, New York, United States of America; 4 Systems Medicine in the Department of Pediatrics, Stanford University School of Medicine, Stanford, California, United States of America; 5 Department of Psychiatry, Duke University School of Medicine, Durham, North Carolina, United States of America; 6 Institute for Health Informatics, University of Minnesota, Minneapolis, Minnesota, United States of America; 7 Department of Biostatistics, Vanderbilt University, Nashville, Tennessee, United States of America; University of Stellenbosch, SOUTH AFRICA

## Abstract

Conventional research methodologies and data analytic approaches in psychiatric research are unable to reliably infer causal relations without experimental designs, or to make inferences about the functional properties of the complex systems in which psychiatric disorders are embedded. This article describes a series of studies to validate a novel hybrid computational approach–the Complex Systems-Causal Network (CS-CN) method–designed to integrate causal discovery within a complex systems framework for psychiatric research. The CS-CN method was first applied to an existing dataset on psychopathology in 163 children hospitalized with injuries (validation study). Next, it was applied to a much larger dataset of traumatized children (replication study). Finally, the CS-CN method was applied in a controlled experiment using a ‘gold standard’ dataset for causal discovery and compared with other methods for accurately detecting causal variables (resimulation controlled experiment). The CS-CN method successfully detected a causal network of 111 variables and 167 bivariate relations in the initial validation study. This causal network had well-defined adaptive properties and a set of variables was found that disproportionally contributed to these properties. Modeling the removal of these variables resulted in significant loss of adaptive properties. The CS-CN method was successfully applied in the replication study and performed better than traditional statistical methods, and similarly to state-of-the-art causal discovery algorithms in the causal detection experiment. The CS-CN method was validated, replicated, and yielded both novel and previously validated findings related to risk factors and potential treatments of psychiatric disorders. The novel approach yields both fine-grain (micro) and high-level (macro) insights and thus represents a promising approach for complex systems-oriented research in psychiatry.

## Introduction

There is an astonishing array of advances in areas such as genomics, proteomics, and functional brain imaging that can provide detailed information about the nature of psychiatric disorders. The capacity to integrate this knowledge with information related to phenotypic expression in the form of cognition, emotion, and behavior, and information related to developmental and environmental risk, opens new frontiers for understanding the nature of psychiatric disorders. These opportunities will not be harvested, however, without the development and evaluation of new computational methods and tools needed for integrating the diversity of modalities of information (e.g. genes, proteins, brain circuitry, developmental, social, behavioral) into unified analyses. Conventional methods and techniques cannot “see” the complex substrate from which psychiatric disorders emerge and are sustained, which is not only based on the relations between variables that span the aforementioned diverse modalities of information, but on the emergent properties of the system which these variables, and their relations, create. If the *whole* of knowledge related to the nature of psychiatric disorders is greater than the *sum of its parts*, then advances in our science will require methods to generate knowledge about both the *parts* and the *whole*.

This article describes a computational method and set of analytic tools that we have developed for these purposes. Obviously, our goal for achieving this method is challenging both conceptually and technically. A successful method will need to be able to accomplish two interrelated tasks:

1. Yield knowledge of the behavior of the complex system (e.g. psychiatric disorder) itself: This knowledge will need to develop from methods that can provide meaningful information about the behavior of the system as a whole, including how the system and its components emerge and are sustained over time. Such methods will need a successful approach for integrating diverse forms of information. Information limited to the separate components of the complex system is unlikely to converge on knowledge about the behavior of the complex system as a whole. Although research has been conducted on several of these components such as brain, gene, and phenotypic networks [1–10], the development and employment of methods to handle the integration of diverse forms of information (e.g. molecular, brain circuitry, developmental, social, behavioral) in a consistent, and meaningful, fashion will be very important.
2. Yield knowledge about mechanisms related to the interactions between various components of the complex system: A complex system, when viewed as a whole, has emergent properties that support its functioning and adaptation over time. It also has components that interact in the form of mechanisms related to its functioning. Mechanistic understandings are causal. Conventional research methods and data analytic techniques are unable to reliably infer causal relations without randomized experiments, and randomized experiments are usually unfeasible and/or unethical in psychiatric research. Research that aims to integrate the diversity of information required to identify the emergent behavior of the complex system governing psychiatric disorders will inevitably identify many relations that are non-causal and therefore non-mechanistic. Accordingly, there is a need for accurate methods to enable inferences about the *causal relations* between variables, or sets of variables, within the complex system sustaining psychiatric disorders.

Accomplishing the two aforementioned tasks in a unified analytic approach poses non-trivial challenges. We intend for the method described in this article to contribute to the development of a diversity of approaches to meet these challenges. Fortunately, the last two decades has seen a great deal of progress in two fields that offer much promise to meeting these challenges: Network Science and Causal Discovery. While a wide range of names has been used for the sets of methods employed in each of these respective fields, for purposes of clarity and consistency, we use the terms *Network Science* and *Causal Discovery* to refer to these respective fields and sets of methods. Specifically, methods have been developed within the field of Network Science that enable the generation of knowledge about the behavior of the system in which complex phenomena, like psychiatric disorders, manifest. Investigators employing the methods and techniques of Network Science have found remarkable consistency in the properties of a broad array of systems that are adaptive and robust in nature [9–17]. Systems that exhibit these measurable properties are called Complex Adaptive Systems (CAS) [13]. The application of Network Science to problems of health and disease is called Network Medicine and its main idea follows: a disease represents a pathologic biological process that emerges, and is sustained over time, because it is embedded in a transformed biologic system that acquires adaptive properties [9–10,14–16]. Accordingly, if such an adaptive system related to a given disease is identified, the capacity to determine its areas of vulnerability may reveal promising targets or new approaches for treatment. Similarly, methods have been developed in parallel in the field of Causal Discovery that employ a family of models (Causal Probabilistic Networks and variants) to infer causal relations from observational data [18–22]. Very recently, algorithms that infer such causal relations with very large numbers of variables have been developed and several empirical studies have verified their applicability [20–21]. Interestingly, although the Network Science and Causal Discovery fields have each generated methods with promise for addressing the two identified challenges for psychiatric research, they have largely developed in parallel with little integration of their methods.

We have created a method and set of computational tools to integrate Causal Discovery within a Network Science framework for psychiatric research. Our approach enables *both* complex and causal systems inference in a unified analysis so that psychiatric investigators can understand the properties of the systems in which psychiatric disorders emerge and are sustained, and search these systems for causal information about their most critical points of vulnerability. We refer to this set of methods and techniques as the Complex Systems-Causal Network (CS-CN) method. This method is designed to transform most any dataset related to psychiatric research for such integrated analysis by performing three overarching computational operations: (1) create a causal network by examining the possible causal association between each pair of variables in the dataset, using the framework of local causal graph, Markov Boundary induction and local-to-global causal discovery algorithms; (2) search the causal network for sets of variables and relations that–when examined together–reveal properties consistent with a CAS; and (3) determine the specific points of vulnerability in the identified CAS. Details on these three computational operations are provided in the Methods section.

Network Science and Causal Discovery have been used in limited ways in psychiatric research, largely related to exploring *either* the causal relations between sets of variables in limited domains (e.g. causal connections between brain regions for specific areas of psychopathology [23–25]) *or* network structure of sets of variables in similarly limited domains (e.g. properties of specific brain networks, molecular networks, or disease phenotype networks for specific areas of psychopathology [1–7]. Our integrated CS-CN method considerably advances the use of these methods for psychiatric research in the following ways: (1) it enables the integration of any modality of information (e.g. molecular, developmental, neurologic, phenotypic, social) in a unified analysis; (2) it integrates state-of-the-art causal discovery algorithms to search an identified network for the causes of psychiatric disorders; (3) it extends these causal discovery algorithms in a unique way–to search for the variables that disproportionately contribute to the identified system’s adaptive qualities. This article presents a series of studies that apply the CS-CN method for psychiatric research and assesses its validity and utility.

## Methods

This study used deidentified data from preexisting datasets collected by the lead author for other purposes. Due to the deidentified nature of the data used in this study, we received exempt status from the IRB at New York University School of Medicine. Our IRB record number is 11–00293.

We first describe the CS-CN method and apply it to an existing dataset on risk factors for psychopathology in 163 children hospitalized with injuries (validation study). Next, we apply it to a large, independent dataset of traumatized children (replication study). Finally, we conduct a validation experiment, under controlled conditions of the CS-CN method–compared to other methods–for accurately identifying causal variables, using a ‘gold standard’ dataset for causal discovery (resimulation controlled experiment).

**Fig 1:**
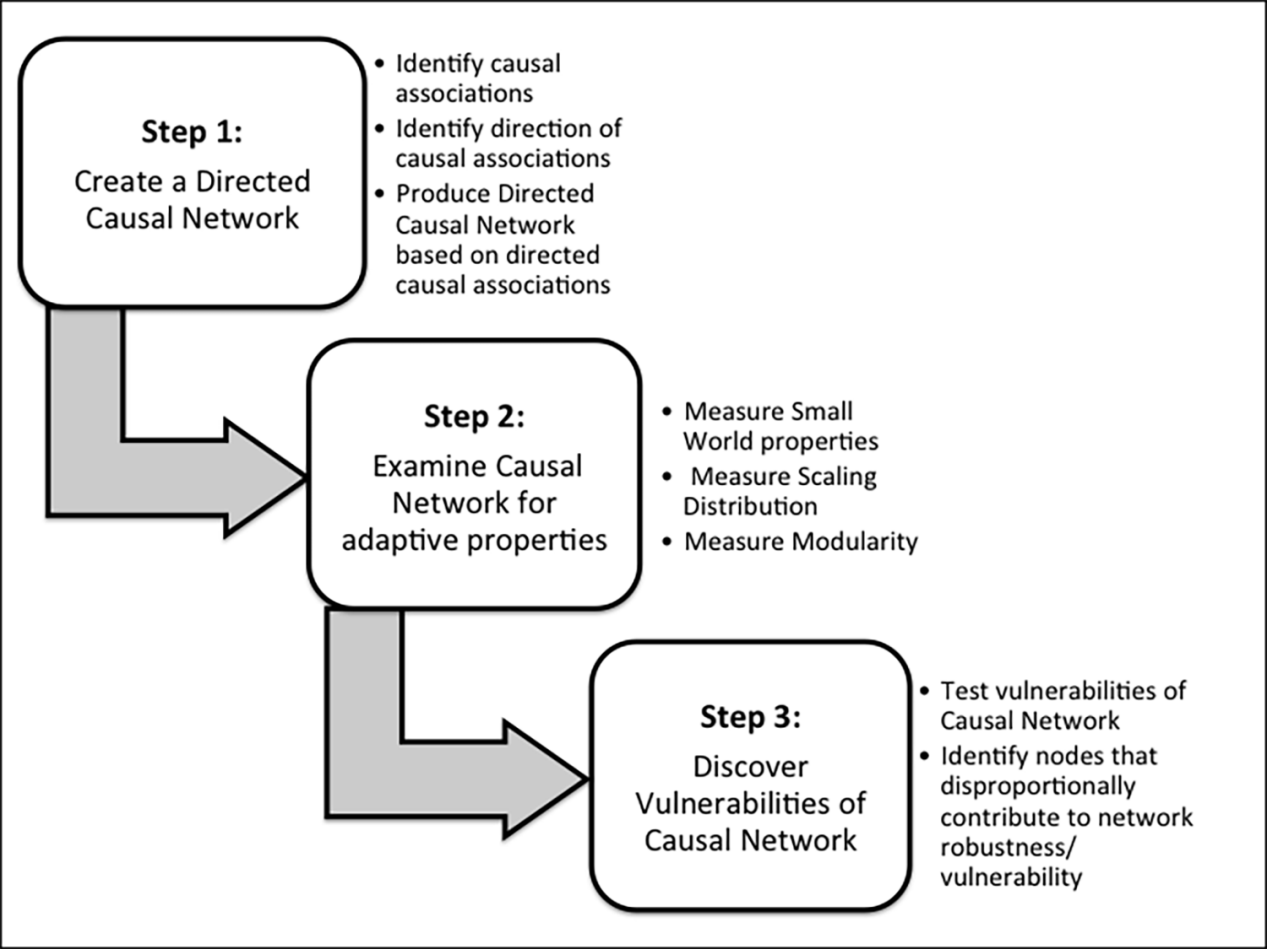
The Complex Systems-Causal Network (CS-CN) Method.

The CS-CN method includes a defined data preparation approach to enable its use. Once this approach is followed, the CS-CN method will process and analyze a dataset in three sequential steps. These steps are illustrated in Fig 1.

### Data Preparation

The CS-CN method is designed so that most any dataset collected for psychiatric research can be uploaded to a web-based platform we created for processing and analysis. Datasets can be automatically processed when uploaded with an additional Variable Table that contains specific information, provided by the investigator, about each variable in the dataset. The information required in the Variable Table for automatic processing includes: the *construct* the variable measures, the nature of its *numerical value* (e.g. continuous, categorical), the *time* époques in which it exerts its effect, and its possible *hierarchical nature* (e.g. a ‘subscale’ value or a ‘total’ value from a psychometric instrument; a brain region, a brain circuit, or a neuron within that region). (We provide detail in S1 File about the information investigators must provide in the Variable Table). Data processing procedures are currently implemented in the Python language [26] while causal graph analytics algorithms are implemented in Matlab [27]. Together, these tools form the web-based platform, which is comprised of a graphical user interface that processes this information in three overarching computational steps that define the CS-CN method.

### CS-CN Method

**Step 1:** *Create a directed causal network by examining the possible causal association between each pair of variables in the dataset using the framework of causal graph and Markov Boundary induction algorithms (****Microstructure Analysis****)*.

a. Identifying causal associations: The CS-CN method considers variables within a dataset as ‘nodes’ and bivariate relations as ‘links,’ and establishes a network of nodes and links that are all consistent with well-defined mathematical properties of causality [18–19]. The relationships between all possible bivariate relations in the dataset are examined and a binary decision (0 or 1) is made about whether a given bivariate relation is *causally-consistent* (1) or not (0). Informally, we call a statement of the type “A is directly causing B” *causally-consistent* if the statistical dependencies and independencies in the data interpreted by the mathematical theory of causal induction from non-experimental data suggest that (a) A and B are not confounded by a measured variable (and in special circumstances even when data contains hidden variables) and (b) the causal relationship between A and B is not mediated by intermediate measured variables. The mathematical theory and corresponding algorithms that enable such inferences were pioneered by Simon, Pearl, Granger, and colleagues [18–19, 28–30]. All causally-consistent bivariate relations are included as links in the network. All other bivariate relations are excluded. This decision is made through application of the framework of causal graph and local causal induction algorithms from the Generalized Local Learning (GLL) and Local to Global Learning (LGL) families [18–22]. This framework allows the application of the aforementioned causal discovery methods [18–19, 28–30] to a scale up to millions of variables (whereas the original algorithms could not handle efficiently more than ~100 variables). Bivariate relations found to lie within the Markov Boundary contain the direct causes, direct effects, and direct causes of the direct effects of the response variable and thus are locally *causally-consistent* but at the same time have desirable predictive and diagnostic properties (i.e. the Markov Boundary is for the majority of distributions the minimal set of variables that contains the full predictive information of the whole data for a response variable of interest [21, 29].

b. Identifying the direction between associations: Our method orients all links in the network to create a *directed* causal network via two types of procedures.

First, links are oriented from information that is provided in the aforementioned Variable Table about the time époques in which a variable may have exerted its effect. Any link connoting a bivariate causal relation that contains variables from different time époques is oriented from the earlier to the later time époque (with the assumption that causal influence must travel forward in time). Investigators will need to make decisions about how variables within their datasets should be divided by time époque classification using the following formal definition: A time époque represents a slice of time, more formally a time interval [t1, t2] where t is a time point in any conventionally constructed real-numbered timeline (i.e. as comprising the set of the real numbers each one uniquely corresponding to a time point in the real world), and where t2-t1 is equal to a pre-defined duration of an époque (a month, a year, or other as defined by the analyst); within this époque (time slice) the directed causal link between variables ‘A’ and ‘B’ (A→B) normally has the following temporal semantics: ‘A’ occurs within time interval [A_start, A_end] which is a subset of [t1, t2], ‘B’ occurs within time interval [B_start, B_end], and: A_end ≤ B_start [31–34].

Second, the remaining undirected causal bivariate relations can be further oriented using a variety of approaches including identification of “Y structures” and constraint propagation [19, 28], Bayesian search and score [30,35], or heuristic approaches [36–37]. In the present application of the method we use the technique by Guo, Yang, and Zhou [36] primarily because it is mathematically convenient for handling directionality within a mixed data type network. This method leverages information about *known* directionality of some of the links for orienting the directionality in the others. Nodes in a directed network are defined by their out-degree value (the number of directed links that point *from* the given node *to* other nodes) and their in-degree value (the number of directed links that point *to* the given node *from* other nodes). Accordingly, causal influence in a directed network flows from nodes with higher out-degree and to nodes of higher in-degree. This method employs information that is known about the directionality for each node (i.e. the node’s indegree and outdegree value), and ranks nodes with this information through a recursive process. Undirected links are then oriented via the differential ranking of each of the two nodes they join. Details of this method are found in Guo, Yang, and Zhou [36]. While the method is highly heuristic and thus does not always guarantee correctness its empirical performance in a study of four diverse existing directed networks where directionality was known for each link showed good ability to orient these links with a very high degree of accuracy [36].

This examination of the presence of *link* between all pairs of variables in the dataset, and the *directionality* between them, creates the *directed* causal network required for steps 2 and 3 of our method.

**Step 2:** *Examine the causal network for sets of variables and relations that–when examined together–reveal properties consistent with Complex Adaptive Systems (CAS)* ***(Macro Structure Analysis)*.**

At this point, we have completed the CN part of the CS-CN method. A directed causal network has been produced, comprising bivariate relations that are all causally-consistent. The next step is to analyze it for its adaptive properties (and visualize it for the benefit of the researcher). If the network demonstrates certain adaptive properties–such as scaling, efficiency of information flow, modularity, and robustness–it is considered a CAS [11, 13–17]. If such properties are observed in a causal network containing psychopathology, it supports the notion that the set of variables–and the way they operate within the system–have contributed to the emergence and maintenance of the psychopathology in question. This second step serves as a validity check for the method. If the causal network revealed in the first step does not have adaptive properties–assessed in the second step–then there is no reason to proceed to the third step. Further, if the CS-CN method is not able to reliably identify causal networks with adaptive properties, the validity and/or utility of the method or the data should be questioned.

The CS-CN method is designed to analyze the network properties of the causal network produced in Step 1 and to compare these properties to their mean values derived from 1000 permutations of a random directed network model. This random directed network model is obtained via the generation of 1000 permutated networks, each with the same number of nodes and links as the observed directed causal network and with the link direction between each pair of nodes in each of these random networks set at chance. The network properties of the random directed model used by the CS-CN method is calculated as the mean value of each respective network property from these 1000 permuted random networks. Table 1 describes the network properties assessed by the CS-CN method and the adaptive nature of each property, as observed in the literature. S3 File provides details on the calculation of each of these properties. Our application integrates Matlab BGL [38] to derive each of the network properties described in Table 1 and detailed in S3 File. The comparison random network models were generated using the modified version of Matlab Tools for Network Analysis [39]. Network visualization is conducted with the Cytoscape platform [40] and cluster analysis and visualization is conducted with the Cytoscape ClusterOne module [41].

**Table 1:** Definitions of network properties, their adaptive qualities, and differences between random networks and adaptive networks [15–17].

Network Property	Definition	Adaptive Quality	Random Network vs. Adaptive Network
Network Diameter	The greatest distance between any pair of nodes in a network. This is equivalent to the longest, shortest path within the network.	Efficiency of information transfer is related to the number of steps it takes to get from one node to another within a network. Accordingly, a larger network diameter is an index of a less efficient network.	A random network has a larger network diameter than an adaptive network.
Characteristic Path Length	The average (mean) number of steps it takes to get from any two nodes in a network along the *shortest path* between those nodes.	Characteristic Path Length is a defining feature of the ‘small world’ property of an adaptive system. It describes the number of steps between any two nodes in the network and, like network diameter, is an index of the efficiency of information transfer within a network.	A random network has a larger characteristic path length than an adaptive network.
Shortest Path Distribution	A ‘shortest path’ between any two nodes is defined as the path with smallest number of steps between those two nodes. The Shortest Path Distribution shows the distribution of Shortest Paths length in the network	A Shortest Path distribution of an adaptive network, like its Characteristic Path Length, illustrates the efficiency of information transfer within the network by showing the small number of steps it takes for information to travel between any two nodes in the network.	Shortest paths in a random network has greater mean path length than an adaptive network. The shortest path distribution of an adaptive network has a higher frequency of very short path lengths.
Degree Distribution	The distribution of number of links per node within the network. Particularly important is the scaling property of the degree distribution defined by the exponential function of the distribution in the equation: *y* = *β* *x*^*α*^	Entities/variables/nodes within an adaptive network do not link by chance, but for functional reasons. Accordingly, an adaptive network displays ‘preferential attachment’ between nodes. This can be observed by the power function of the link-per-node distribution.	A random network displays a normalized link-per-node distribution. An adaptive network is ‘scale free.’ Most nodes draw few links while a small number of nodes draw an extraordinarily large number of links.
Clustering Coefficient	A network’s clustering coefficient is an index of the degree to which the network contains regions of nodes that are highly interconnected.	A network’s clustering coefficient indicates the possible modular nature of a network. An adaptive network tends to be modular as different parts of the network assume different specialized functions.	A random network has a smaller clustering coefficient than an adaptive network.

The CS-CN method inserts one process of data preparation before conducting the network analysis of the causal network produced in Step 1. This process is designed to safeguard the analysis from falsely demonstrating adaptive properties based on measurement biases related to the type of data under study. Such biases can intrude into the analyses based on the ubiquity of what might be called *structurally superfluous information* within typical datasets for psychiatric research. We have created procedures for removing superfluous information from the directed causal network identified in Step 1. These procedures are detailed in S2 File.

Importantly, the CS-CN method allows for assessing the network properties of the entire identified causal network, or to search the causal network for sub-networks that may have adaptive properties. There are two reasons for conducting such a search: (1) the global causal network itself is highly heterogeneous masking the properties of the sub-network components that are themselves CASs, and (2) there is a scientific rationale for searching for a sub-network (e.g. interest in a sub-network related to specific genes or specific brain regions; or interest in separate analyses for different forms of psychopathology contained within a larger causal network). Any sub-network within a larger causal network contains links that are all *causally-consistent* and, accordingly, is a proper causal network. The capacity to use the CS-CN method to conduct such searches provides investigators with a highly flexible and powerful platform for causal discovery. Thus, at the end of this step we have a set of variables that constitutes what we define as a *Complex Systems-Causal Network*. All links in this network are consistent with well-established causal assumptions, and the network has properties consistent with CASs.

**Step 3:** *Discover the vulnerabilities of the Complex Systems-Causal Network to generate knowledge related to pathogenesis*, *treatment targets*, *and high-level functional organization* ***(Functional Inference Analysis)*.**

Once the macro/adaptive properties of the Complex Systems-Causal Network are analyzed, the capacity to search for its points of vulnerability is straightforward. Nodes vary in their contribution to a network’s adaptive properties, and there are a variety of ways to identify a node’s importance to the adaptive properties of a network (defined as the node’s *centrality*). The simplest way of defining the centrality of a node is to count the number of other nodes that link to it (defined as *degree centrality*). Another index of the importance of a node to a network is called *betweenness centrality* (BC), defined as the number of shortest paths that pass through a node. BC is thought to reflect the amount of control that a node may exert over other nodes in the network. Given the importance of BC, we use it as our primary index of a node’s centrality. The conventional method for assessing the vulnerability and robustness of a network to challenge is to model changes in the adaptive qualities of a network to node removal by centrality rank vs. randomly generated rank [11–13]. An adaptive network loses its adaptive properties when its most central nodes are sequentially removed. An adaptive network is highly robust to challenge by random node removal. This process of ‘error tolerance vs. attack vulnerability’ is thought to be an important quality of a Complex Adaptive System (CAS) [11–13]. The modeling of these forms of challenge offers two advantages: 1. The results will reveal whether the network ‘behaves’ consistently with other CASs to challenge, thus supporting (or refuting) a conclusion that our method has identified a CAS related to a psychiatric disorder of interest, and 2. The results will identify the variables (nodes) that most contribute to the robustness of the identified CAS, and may also reveal a critical threshold of node removal before the CAS loses its adaptive properties. The identification of such variables (and the threshold number for removal) may offer important opportunities for intervention development. Our method uses the approach of sequential node removal based on BC rank (with recalculation of rank at each sequential step). As Holmes and colleagues have reported, this approach is amongst the most powerful means for challenging an adaptive network [12]. There are also other methods, found in other literatures, for network challenge (such as making “in silico experimentation” inferences about the quantitative effects of intervention to the network, via, for example Pearl’s “Do Calculus” [19]). In the future we may integrate these additional approaches within our CS-CN application.

### Procedures for Handling High Dimensional Information

As psychiatric research advances, investigators will increasingly need to integrate procedures for handling high dimensional information within their datasets. This has certainly been a challenge for research using more conventional statistical methodologies, related to limitations in the number of variables that can be included in a given analysis. It is also a challenge with the CS-CN method for reasons specific to network analysis itself. In this section, we describe this challenge and detail how the CS-CN method has been programmed to handle it.

A primary concern with the integration of high dimensional information of one modality with information of another modality, within a network analysis, is that measurement biases can influence the study results. If a dataset contains a very large, and disproportionate, number of variables of a given modality (e.g. genes) compared to variables of other modalities (e.g. demographics), the observed scaling properties of the network may simply be driven by the disproportionate number of variables within the high dimensional modality, compared to other variables in the dataset. An important innovation of the CS-CN method is its capacity to integrate a great diversity of modalities of variables into an integrated analysis. Therefore, handling the problem of proportionality between different modalities of information is extremely important.

We handle this problem by using information about the hierarchical nature of variables that are specified by the investigator in the Variable Table, prior to any analysis. Depending on the specific research question and the nature of the high dimensional information in the dataset, investigators can create specific hierarchies from their high dimensional data and specify these hierarchies in the Variable Table. The CS-CN method then uses that hierarchical information to constrain the number of variables that will be used in a singular network analysis. For example, our validation study (described next) contains one sort of high dimensional data, candidate genes, which include a large number of corresponding single nucleotide polymorphisms (SNPs). The CS-CN method is programmed to conclude that if any variable of a lower hierarchy (e.g. a SNP) is found to be causally related to any other variable in the dataset, its higher-order variable (e.g. its gene) is considered to be causally related to that variable and only that higher order variable is described in a network analysis. Although we have described this problem (and our solution) concerning genomic information, the CS-CN method can handle this problem with any form of disproportionality related to high dimensional information within a dataset. We expect our solution will be very relevant for handling high dimensional brain imaging information in an integrated analysis, related to the hierarchical nature of this information (e.g. voxels, brain regions, brain circuits).

### Confirmation Studies

#### I. Validation Study

The Child Injury Dataset (CHIDS) is based on 163 children aged 7–18, and was collected as part of a National Institute of Mental Health funded study (R01 MH063247) on risk factors for psychopathology in children hospitalized with injuries. The basic design follows: injured children were assessed within hours or days after their hospitalization and reassessed 3 months and 1 year following discharge. CHIDS includes 161 variables related to such domains as candidate genes, early childhood development, demographics, school and social function, family stress, parent symptoms and functioning, psychosocial stress, qualities and magnitude of injury, neuroendocrine response, psychophysiologic response, and child symptoms and functioning. Due to space restrictions, we provide information about each of the 161 variables of the CHIDS in our Variable Table in S1 Table.

#### II. Replication Study

We applied the CS-CN method to a dataset of 14,088 children and 1,439 variables called the National Child Traumatic Stress Network (NCTSN) Core Dataset (CDS) [42]. Details about the methods and results for this replication study are found in S5 File.

#### III. Resimulation Controlled Experiment

Following the standard of rigorous new method validation using model-based simulation (e.g. “resimulation”), the CS-CN method was tested in a controlled experiment using a ‘gold standard’ dataset for causal discovery obtained by sampling a previously published model that has been used extensively for causal challenges and testing of new methods. The ability of CS-CN to discover causal structure was compared with other methods for accurately detecting causal variables against the known model structure. Details of this study are provided in S6 File.

## Results

### Finding the Causal Network (Micro Structure Analysis)

The CHIDS dataset of 161 variables, and its corresponding Variable Table, were uploaded into our web-based CS-CN platform. The causal discovery algorithm identified 129 nodes and 204 links. Of these, 18 nodes and 37 links were considered to be superfluous (by the method for identifying superfluous variables, as detailed in S2 File) and were eliminated. Accordingly, we produced a causal network containing 111 nodes and 167 links.

### Determining the Complex Systems Properties of the Causal Network (Macro Structure Analysis)

**Fig 2:**
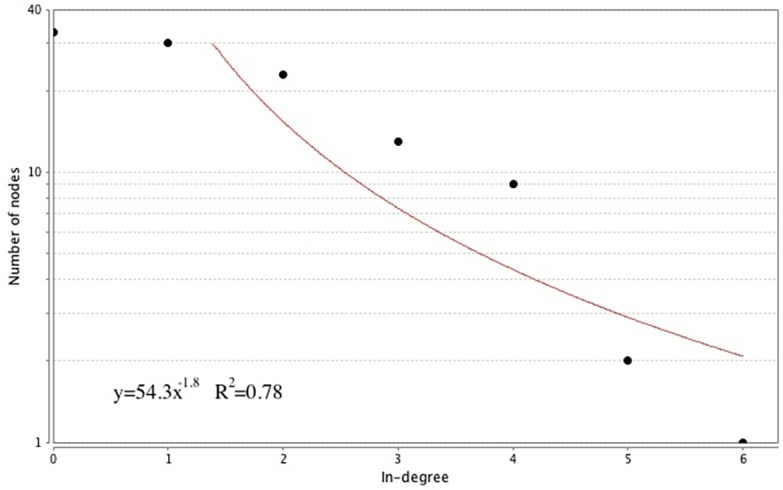
In-degree Distribution of the CHIDS Network (logarithmic scale).

**Fig 3:**
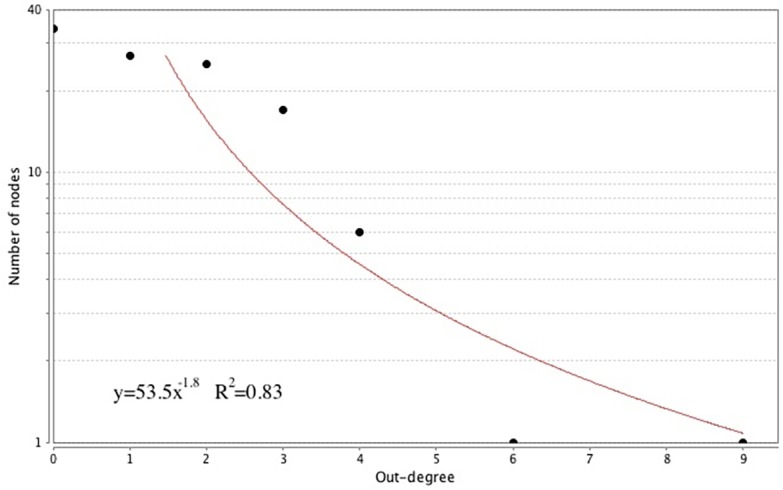
Out-degree Distribution of the CHIDS Network (logarithmic scale).

The 111 node/167 link causal network was analyzed for its complex systems properties. Because the causal network is *directed*, we determined both its *in-degree* and *out-degree* scaling properties, based on the distributions of numbers of links entering and leaving nodes, respectively. In Figs 2 and 3, we show these scaling distributions, with logarithmic transformation.

The properties of this network were compared to the mean value of these properties in a modeled random directed network of 1000 permutations. The random network was derived from the same number of nodes (111) and links (167) as the CHIDS network. The comparison between these networks is shown in Table 2.

**Table 2:** Properties of the CHIDS Causal Network vs. Random Directed Network.

Network Property	CHIDS Causal Network	Random Directed Network (mean of 1000 permutations)
Nodes	111	111
Links	167	167
Network Diameter	5.00	18.11
Characteristic path length	1.86	6.58
Clustering Coefficient	0.05	0.01

As seen in the results in Table 2, the CHIDS Causal Network has very different properties from the Random Network even though they have the same number of nodes and links. The causal network is less than one third the size of the random network (based on diameter and characteristic path length), indicating its ‘small world’ properties–a strong indicator of efficiency of information transfer. It has a clustering coefficient five times as large, indicating its modular functioning. These network properties are consistent with Complex Adaptive Systems.

**Fig 4:**
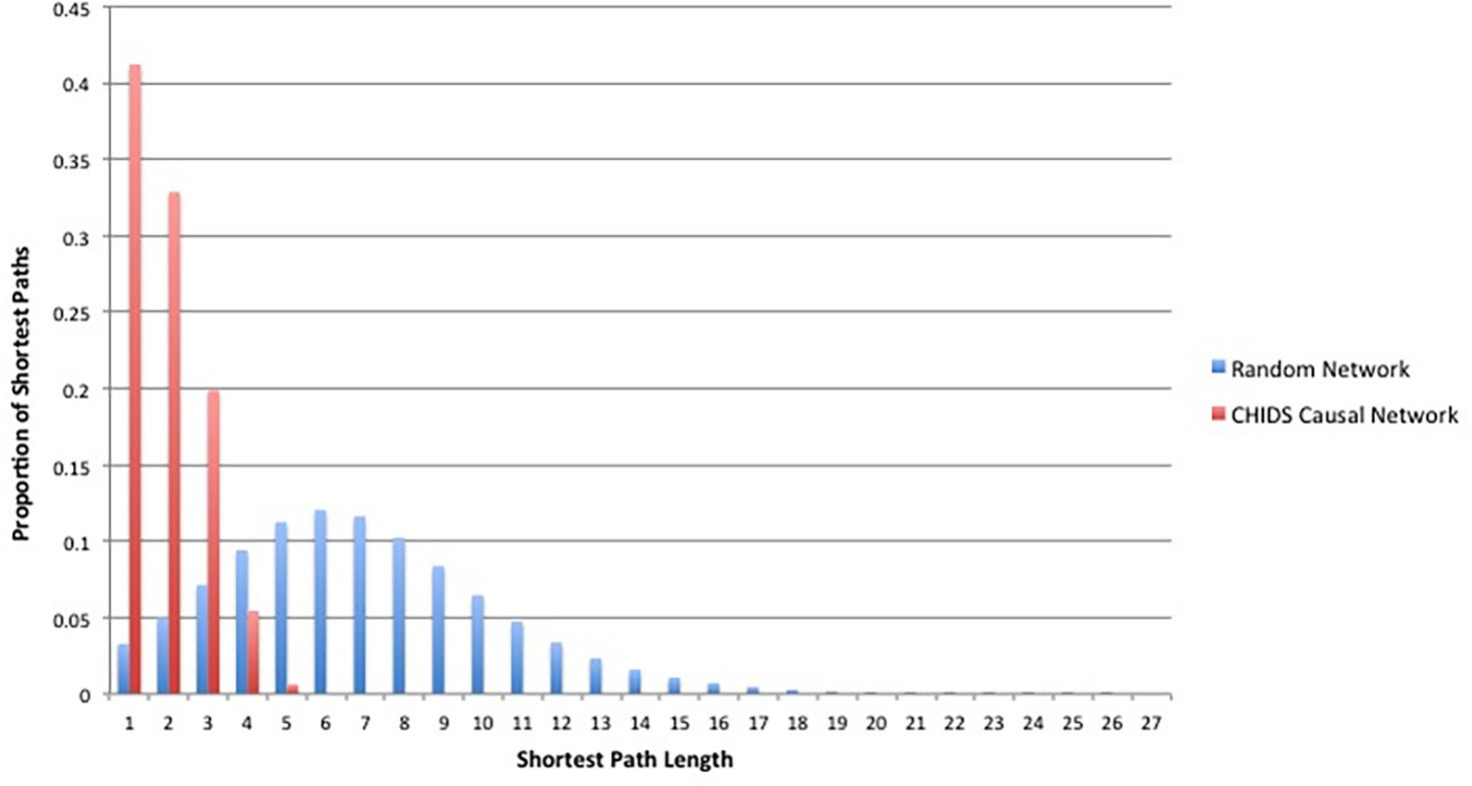
Distribution of Shortest Paths in the CHIDS Network and the Random Network.

The small world-ness of the CHIDS Causal network can be seen clearly in Fig 4, which shows the shortest path distribution of this network vs. the random network. As can be seen, the distribution of shortest paths in these two networks is dramatically different. Over 40% of shortest paths in the CHIDS causal network contain only 1 link and the longest shortest path is only 5 (network diameter). The random network is distributed with only 3% of shortest paths containing only 1 link and the majority of its paths are over 5 links wide, with many considerably wider. We conducted a two-sample Kolmogorov-Smirnov (K.S.) test to determine if these respective distributions were statistically distinct (under the null hypothesis that the two distributions are the same). This analysis confirmed that the distribution of shortest paths of the CHIDS causal network was very different from that of the random network (K.S. = 0.78, p = 4.7 x 10^−198^).

**Fig 5:**
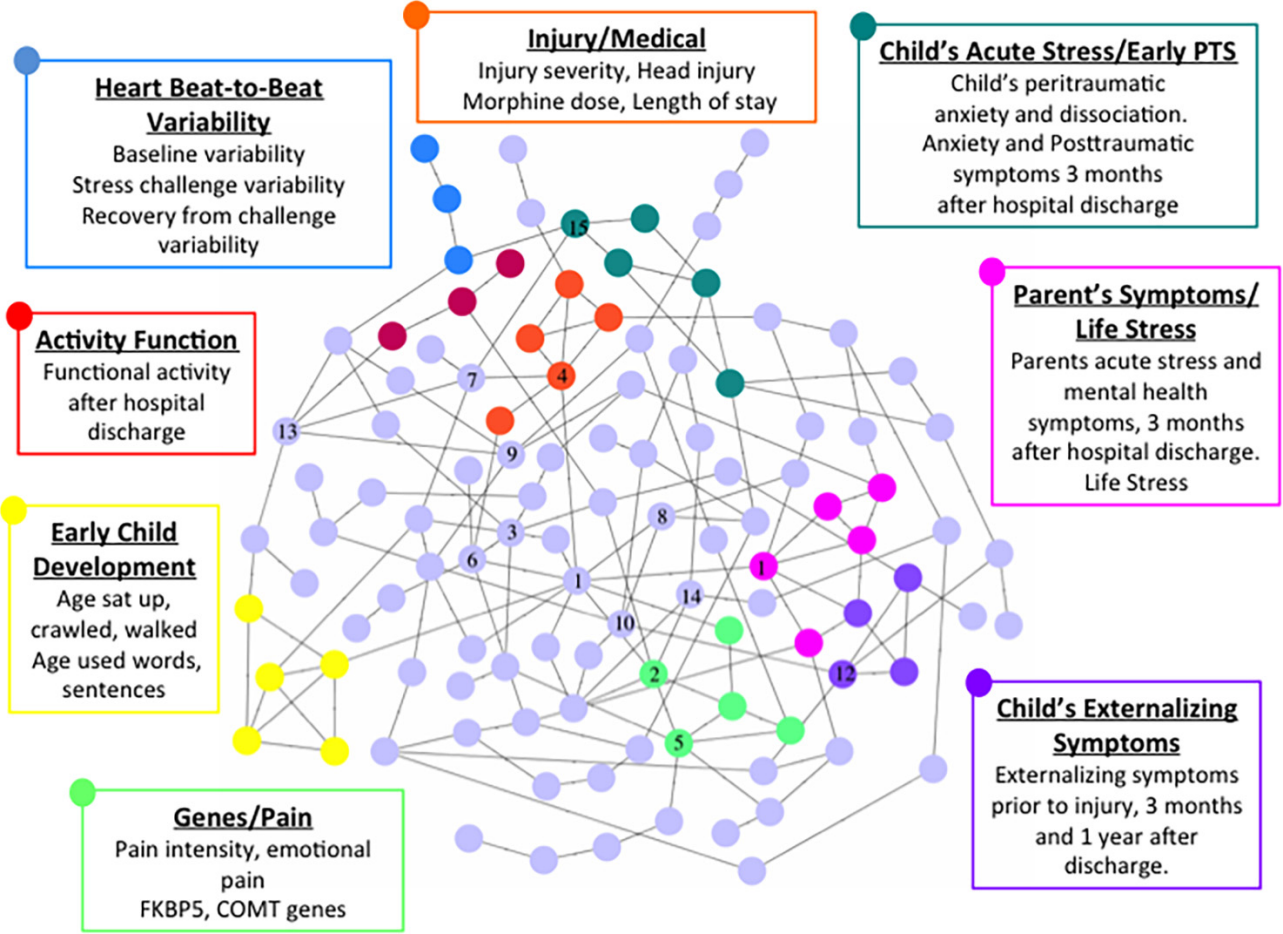
The CHIDS Network and its Eight Modules. The 15 highest ranked nodes based on BC score are indicated by numeric rank order.

We provide a visualization of the causal network and its modular nature in Fig 5. Eight modules were discovered (color-coded) by the ClusterOne plugin to Cytoscape. As can be seen, the variables contained within a given module appear to fit together conceptually and have face validity for modules that can be expected to relate to the development of child psychopathology amongst injured children. We labeled each module based on the type of variables that were contained within it. This labeling can also be automated although in the current version of the software it is manual. We also include the numerical rank order of the 15 nodes with the highest BC scores. Details about each of these nodes, and how they were measured, are provided in Table 3, S1 Table, and S4 File.

**Table 3:** Top Fifteen Nodes by BC rank.

Variable	Betweenness Centrality Score	Measurement
1. CRHR1 Gene	3556.64	9 SNPs on the CRHR gene (CRHR104- rs17763104, CRHR112- rs12944712, CRHR114- rs17690314, CRHR142- rs242942, CRHR144- rs4458044, CRHR158- rs17763658, CRHR161- rs4074461, CRHR181- rs12936181, CRHR192- rs11657992) analyzed via buccal DNA samples obtained via mouthwash, isolated using Gentra DNA isolation kit, and typed using real-time PCR technology.
2. FKPB5 Gene	1881.30	9 SNPs on the FKBP gene (FKBP547- rs3777747, FKBP573- rs3800373, FKBP502- rs4713902, FKBP558- rs9296158, FKBP524- rs9380524, FKBP534- rs10498734, FKBP563- rs10947563, FKBP542- 17614642, FKBP533- rs6926133) analyzed via buccal DNA samples obtained via mouthwash, isolated using Gentra DNA isolation kit, and typed using real-time PCR technology.
3. Social Competence prior to injury	1630.97	Child’s score on the Social Competence scale of the Child Behavior Checklist (CBCL) [43] about the child prior to the injury.
4. Morphine Dose (mg/kg/day)	1626.80	Morphine use (mg/kg/day) during total length of hospital stay as recorded on child’s medical record.
5. COMT Gene	1471.25	2 SNPs on the COMT gene (COMT33- rs4633, COMT69- rs6269) analyzed via buccal DNA samples obtained via mouthwash, isolated using Gentra DNA isolation kit, and typed using real-time PCR technology.
6.Socioeconomic Status	1356.03	Child’s socioeconomic status as captured by the Diagnostic Interview for Children and Adolescents (DICA) [44].
7. Age at Trauma	1351.71	Child’s age in years at the time of trauma.
8. Physical Symptoms of Anxiety @ 1 Year	1294.08	Child’s score on the Physical Symptoms scale of the Multidimensional Anxiety Scale for Children (MASC) [45] 1 year following injury.
9. Happiness and Contentment Before 1 Year Old	1247.23	Child’s happiness as a baby as captured by the DICA.
10. Depressive symptoms @ 1 Year	1041.49	Child’s total score on the Child Depression Inventory [46], 1 year following injury.
11. Internalizing Symptoms Prior to injury	944.84	Child’s score on the Internalizing scale of the CBCL about the child prior to the injury.
12. Externalizing Symptoms at 3 Months Post-Injury	909.85	Child’s score on the Externalizing scale of the CBCL 3 months following injury.
13. Diastolic Blood Pressure	904.95	Mean diastolic blood pressure during entire length of hospital stay as recorded in the nursing record.
14. Activity Function Prior to injury.	904.20	Child’s score on the Activity Competence scale of the CBCL about the child, prior to injury.
15. Anxiety Post-Trauma Acute	836.56	Child’s total score on the MASC in the hospital immediately following injury.

### Modeling the Adaptive Properties of the Network and Searching for its Points of Vulnerability (Functional Analysis)

**Fig 6:**
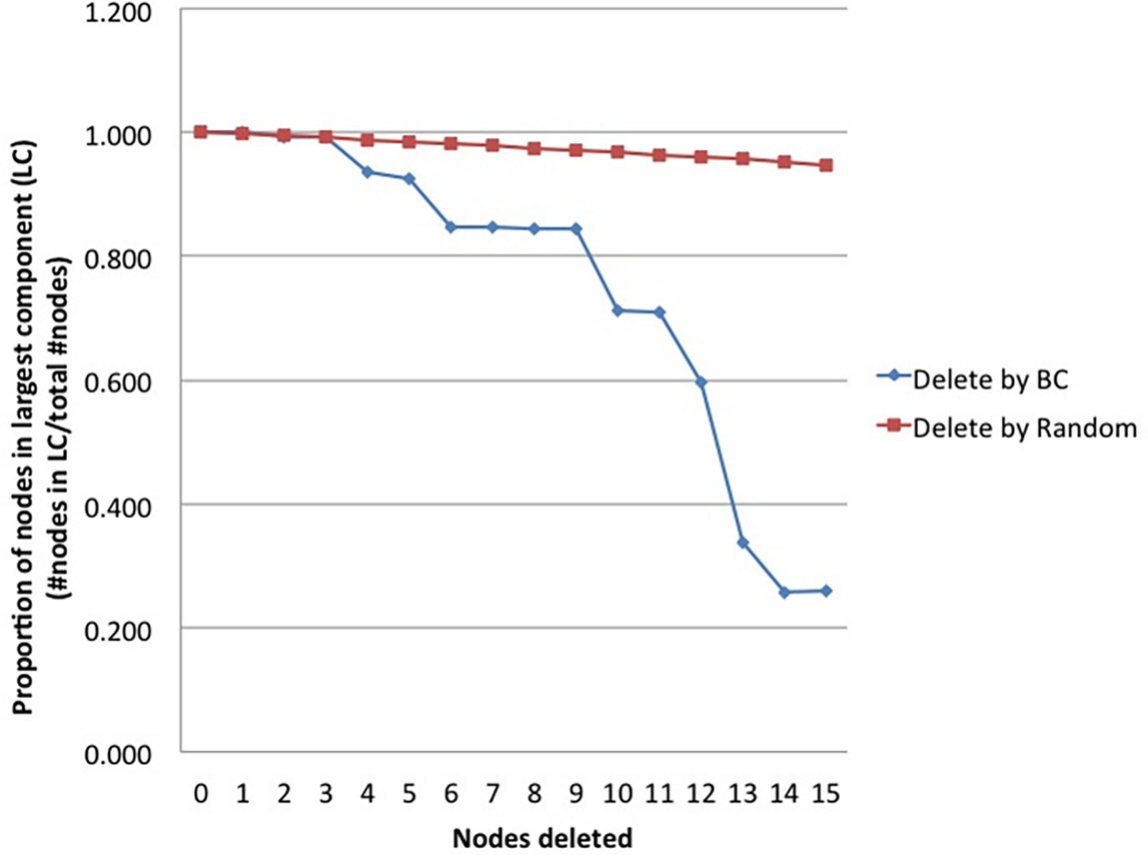
Integrity of CHIDS Causal Network Following Challenge. The proportion of nodes in the largest network component by sequential removal of 15 nodes at random vs. by BC rank.

The final step in our approach is to test the adaptive nature of the network by modeling how it behaves when stressed by the sequential removal of 15 nodes at random vs. by BC rank order. In Fig 6, this differential response to challenge is shown dynamically based on the network’s capacity to maintain its largest component, a strong indicator of robustness. We examine the proportion of nodes that remain in the largest component of the network following sequential removal by rank order based on BC rank or by random number generator (with recalculation of rank at each step) [12]. Each step of the random node removal calculates the mean of the remaining proportion of nodes in the largest component from 1000 trials. As can be seen, random sequential removal of 15 nodes has negligible impact on the network. The CHIDS network maintains close to 100% of its integrity throughout this sequential challenge. On the other hand, the sequential removal of nodes by order of BC rank leads to a considerable loss of network integrity. By the end of this 15 node sequential ‘attack’ the CHIDS network has lost almost 80% of its nodes. As can be seen, its integrity is maintained through the sequential removal of its four highest BC ranked nodes, and then the network quickly loses its integrity with more sequential ‘attacks.’

**Fig 7:**
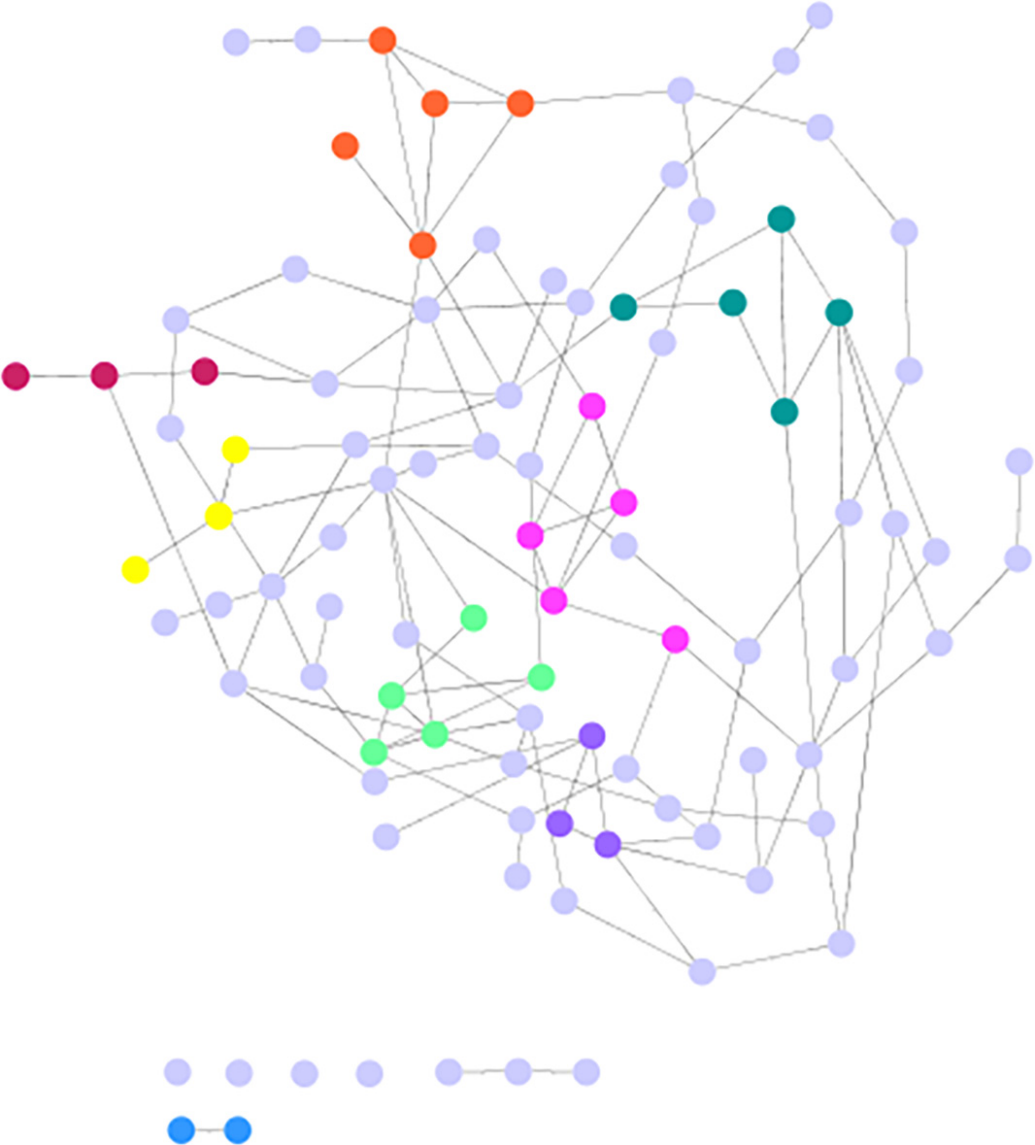
The CHIDS Causal Network After Random Node Removal. The CHIDS network after the sequential removal of 15 nodes at random.

**Fig 8:**
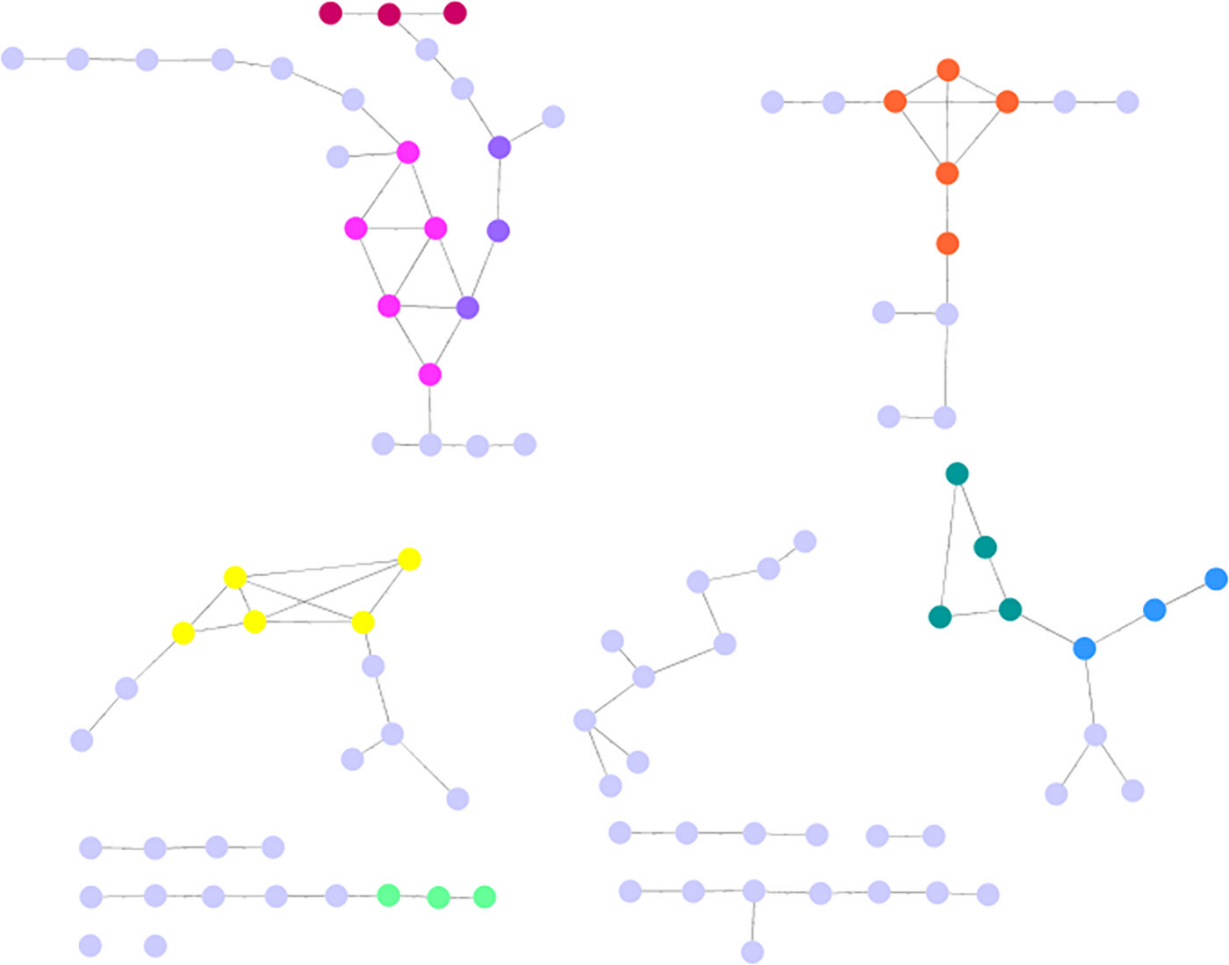
The CHIDS Causal Network After Node Removal by BC Rank. The CHIDS network after the sequential removal of 15 nodes by BC rank.

Another way to visualize the response of the CHIDS network to random vs. targeted (BC rank) attack is by examining the remaining CHIDS network after these two respective forms of challenge are completed, and to compare the remaining network under these two conditions to the CHIDS network before challenge, as shown in Fig 5. The remaining CHIDS network after random removal of 15 nodes is shown in Fig 7. As can be seen, the CHIDS network has largely kept its integrity, losing only 9 of 111 nodes. On the other hand, this same network, when challenged by removal based on BC rank, fragments into 11 components and loses all semblance of its structure (Fig 8).

### A Note on the CS-CN method and Type 1 Errors

One additional advantage of our approach is that, by definition, the variables identified to be most important for sustaining the adaptive properties of a network will have extraordinarily low probability values by chance. The problem of Type 1 errors is ubiquitous in psychiatric research and the ease of finding an erroneous statistically significant result in typical datasets with large-scale hypothesis mining is often not given due consideration. Recently, the phenomenon of publishing erroneous findings based on statistically significant ‘p values’ from datasets of a great many variables has been called ‘P-Hacking,’ and has been offered as a reason for the non-reproducibility of findings in research related to mental illness [47–48]. The CHIDS dataset, for example, contains 161 variables. Accordingly, this dataset contains 25,760 possible directed bivariate relations, 1,288 of which would be expected to be statistically significant (p < .05) based on chance alone. Our method searched all possible bivariate relations and produced a directed causal network of only 167 bivariate relations. Hence, even if no true relationship exists, the false positive discovery rate is less than 0.006 (167/25,760), which is much lower than a conventional alpha level of 0.05. The reasons why our methodology is extremely robust to false positives under multiple hypothesis testing are quite intricate and directly follow from the properties of GLL algorithms [20]. Their robustness to multiple hypothesis testing is well-documented and we refer the interested reader to that prior work for details [21].

## Discussion

We developed a computational approach that enables and integrates causal and systems level inference in a unified analysis for psychiatric research. In our validation study, the CS-CN method identified a network of 111 variables and 167 bivariate connections that had adaptive properties comparable to those found in other fields [11,13–17]. We identified both the robust nature of this network and its points of vulnerability. Modeling the removal of these points resulted in the network losing its adaptive properties. We were also able to successfully apply our method to a much larger dataset of traumatized children in our replication study (detailed in S5 File). We further demonstrated that our novel hybrid causal discovery/network science approach performed comparably to state-of-the-art Causal Discovery algorithms, and better than more conventional statistical methods, in detecting causal variables from a gold standard dataset. These results demonstrate that the CS-CN method can detect causal variables at the level of state-of-the art Causal Discovery algorithms *and* it provides meaningful information about the properties of the complex system in which these variables are embedded. It thus incorporates both a micro causal structure view and a macro level functional view of a system under study.

From the perspective of traditional statistical analytics that have dominated psychiatric research for decades, there is an inherent challenge to proposing a new data analytic method that deviates considerably from established ones. If the findings yielded by the new method are too consistent with what has been reported in the literature, the new method will fail the *novelty* test. Why go to all the trouble of developing and validating a new method if the findings yielded are not *novel-enough*, given the current state of the literature? On the other hand, if the findings derived from the new method deviate too much from what is known from the literature, the new method risks being dismissed out of hand. Accordingly, new methods must achieve a balance of being *novel-enough* and *consistent-enough* with the literature. Next, we discuss the evidence for such a balance.

The three of the top four nodes with the highest BC rank in our validation study (the CRHR1 gene, the FKPB5 gene, and the child’s dose of morphine) have all been found by several prior studies by the lead author (GNS) and others to be related to PTSD in this and other datasets, resulting in previous publications [49–53]. These 3 variables have also been related to the development of PTSD by other investigators [54–57]. These findings are certainly consistent-enough with the literature. What makes them novel?

First, results indicated these 3 variables were *causally* related to the development of psychopathology, a conclusion that could not be inferred from any previous study. Second, these variables were shown to be important contributors to the stability of the adaptive system in which psychopathology was sustained, and modeling the removal of these 3 variables, along with 12 others, contributed to the fragmentation of the system. A dynamic analysis revealed a critical threshold of vulnerability to challenge, shown in Fig 6. The system maintained its adaptive properties until its 4 most important nodes were removed, indicating that effective interventions will require a multipronged approach. Third, examining the nature of these variables provides information about what such a multipronged intervention might look like. Each of the 15 variables shown in Fig 2 and Table 3 indicates the potential utility of these variables for different components of intervention, such as *risk factor profile* (e.g. genes, age, socioeconomic status, social competence and internalizing symptoms prior to hospitalization), *early intervention* (e.g. anxiety in the hospital, diastolic blood pressure in hospital, morphine dose in hospital, externalizing symptoms at 3 months), and *discovery of new treatment targets and interventions* (e.g. drug discovery based on the importance of specific genes). Fourth, an intervention approach can be refined with knowledge that these 3 variables, along with others, comprise different modules contributing to the system’s adaptive properties.

The type of knowledge revealed by the CS-CN method for child traumatic stress could have broad relevance for psychiatry. The capacity of the CS-CN method to identify the variables (of whichever modality) that disproportionally contribute to the stability of psychiatric disorders may generate important information related to risk profiles, early intervention strategies or new approaches to prevention or treatment. Certainly, methods that enable causal inference without experimental study designs are broadly applicable in psychiatry, as is the need to derive better understandings of the complex system that may sustain many psychiatric disorders.

### Limitations

The CS-CN method could be further validated and improved in several ways:

1. Verifying its accuracy and utility with additional psychiatric populations.
2. Investigating the optimal number and duration of time époques to use. Time époque definition is important for causal inference, but datasets will vary widely in terms of time-related information contained within them.
3. Evaluating the relative advantage of different procedures to infer directionality between causal relations within a given time époque. As detailed in the method section, we employed a heuristic approach to define directionality between variables from the same time époque [36]. Although this method has demonstrated a high degree of accuracy in previous studies, the evidence for causal directionality is not as strong for links between variables from the same époque vs from different époques. There exist several different approaches in the literature to address this issue. Future studies could compare these different approaches to define the relative advantages of each.
4. Clarifying the best approach for managing structurally superfluous information. As described in the method section, and in S1 File and S2 File, bias can be introduced if investigators include many variables that measure the same construct in a given analysis. As detailed in these sections, the CS-CN method integrates safeguards for this potential problem but future research should seek to define and evaluate the optimal methods for handling structurally superfluous information.

In conclusion, the extent of the value of the CS-CN method will ultimately relate to its longer-term utility to the broader community of psychiatric investigators. Our approach shares the spirit of an exciting initiative within the biomedical sciences called ‘Convergence.’ As Sharp and Langer wrote in an influential editorial: “The next challenge for biomedical research will be to solve problems of highly complex and integrated biological systems within the human body” [58]. *Convergence* calls for the integration of expertise and information from a wide variety of disciplines towards solving complex biomedical problems. Such an initiative will require novel methods to integrate this diverse information in a unified, and meaningful, analysis. We hope that the CS-CN method may contribute to this important approach to biomedical science, and we look forward to sharing our methods, and improving them, based upon their utility to the field.

## Supporting Information

S1 File Preparing a Data Set for Processing by CS-CN Method: The Variable Table. (DOCX)

S2 File Managing Superfluous Information with the CS-CN Method. (DOCX)

S3 File More Detail on Measuring the Complex Systems Properties of the Causal Network. (DOCX)

S4 File The CHIDS Network Variable Table. (DOCX)

S5 File The Replication Study: NCTSN Core Dataset. (DOCX)

S6 File The Resimulation Controlled Experiment: Validating the CN-CS Method in. (DOCX)

S7 File The CHIDS Dataset in Excel and Text formats. (ZIP)

S1 Table CHIDS Sample Variable Table. (DOCX)
