# Supplementary material for: A Complex Systems Approach to Causal Discovery in Psychiatry
Source: PLoS One. 2016 Mar 30;11(3):e0151174. doi: 10.1371/journal.pone.0151174 (PMC4814084; doi:10.1371/journal.pone.0151174)
Supplement: S1 File — (DOCX) [file pone.0151174.s001.docx]

**S1 File: Preparing a Data Set for Processing by CS-CN Method: The Variable Table**

As described in the section on data preparation, the CS-CN method is designed so that most any dataset collected for psychiatric research can be uploaded for processing and analysis. Datasets stored in any format (e.g. SPSS, SAS, Excel) can be uploaded for processing after they are transformed into Comma Separated Value (CSV) format. An additional Variable Table is also uploaded that contains specific information provided by the investigator about each variable in the dataset. Various algorithms programmed within the CS-CN method will use this information to perform operations in Step 1 of the method to produce the directed causal network. The Variable Table includes all variables as rows and 5 categories of information as columns, which are outlined below. An example of a Variable Table used for the Validation Study (the Child Injury Data Set/CHIDS) is provided in S1 Table.

1. Variable Name: The name designated for the variable in the uploaded dataset.
2. Construct Label: A term that succinctly describes, in accessible language, the construct that each variable is supposed to measure. This is the term that will be used to populate the label of any node within the network, so it must be brief and understandable. Many terms used for variables in typical datasets are highly technical and linked to the measures of constructs. The CHIDS dataset, for example, includes a variable labeled *csdctot12mos*. This variable describes the construct of Post-Traumatic Stress Disorder at 12 Months after hospital discharge as measured with the Child Stress Disorder Checklist (CSDC), an observer report measure of PTSD in children. The construct chosen for this variable is *PTSD12M*. Investigators will need to use discretion about the choice of construct label for the visualization of their network to be understandable to those not familiar with the dataset, and for the CS-CN method to be able to manage the superfluous information in the dataset related to multiple variables that describe the same, or almost the same, construct. The rules for handling superfluous information are detailed in S2 File. The capacity for the CS-CN method to manage superfluous information will be dependent on the clarity with which investigators label their constructs. In the CHIDS dataset, for example, another variable is labeled *ptsdritot12mos*. This variable describes the construct of Post-Traumatic Stress Disorder at 12 Months after discharge as measured with the UCLA PTSD Reaction Index (UCLA PTSD RI), a child report measure of PTSD. The construct label chosen for this variable, like for the *csdctot12mos*, is *PTSD12M*. By labeling variables that measure highly similar constructs with the same construct label, the CS-CN method will select only one using the rules detailed in S2 File. As will be described, variables that are meant to measure the same construct from different observers should be labeled as the same construct. Variables meant to measure the same construct at different time points are labeled differently, with construct name and time designation. Accordingly, variables in the CHIDS dataset labeled as *csdctot3mos* and *ptsdritot3mos* were both given the construct name *PTSD3M*.
3. Numerical Type: Some variables in a dataset will be described with continuous numbers, and others will be categorical. The CS-CN method examines each pair of variables within the dataset and, using causal discovery algorithms described in the methods section, determines if they are related. The algorithms require information about the numerical nature of the variable to perform this operation.
4. Time Epoch: Information regarding the time at which a variable is presumed to exert its effect is used to determine the directionality of each link. Investigators should distinguish a set of clear time epochs in which variables would be expected to exert their effects. The most straightforward way of indicating time epoch is simply the point at which the variable is measured in longitudinal studies. There is also value in specifying a wider range of time epochs if there is strong theoretical or empirical rationale for why a given variable may exert its effect at another point in time from when its measurement occurred. The CHIDS dataset, for example, was collected longitudinally at 3 points in time (acute hospitalization, 3 months and 12 months after hospital discharge) but contains variables that can be expected to exert their effects at other points in time (e.g. the child’s prenatal and early developmental period, trauma before acute hospitalization, etc.). As will be detailed in S4 File, the CHIDS dataset uses 8 time epochs (constitutional, pre-natal, early development, pre-trauma, trauma, acute hospitalization, 3 months post-trauma, 12 months post-trauma). The CS-CN method uses information about time epoch to label bivariate relations from an earlier to a later epoch as directed. Bivariate relations from the same time epoch are not directed.
5. Variable Hierarchy: There are sets of variables within a dataset that have a clear hierarchical structure in that information contained within variables of higher order is comprised of the information contained within variables of lower order. This is most easily seen with psychometric measures wherein items, subscales, scales, and total scores form such hierarchies; it can also be seen in a variety of other sets of variables (e.g. neurons, brain circuits, brain regions). The CS-CN method uses information about the hierarchical nature of specific sets of variables to manage the problem of superfluous information, described in S2 File. Investigators are expected to identify sets of variables within their datasets that form specific hierarchies and, within the Variable Table, provide a numeric designation of the hierarchical level of the variables that fall within the given hierarchy. The CHIDS dataset, for example, used the Child Behavior Checklist as an observer report measure of psychopathology in children. Three hierarchical levels are selected for variables measured by the CBCL: total score (level 1), broad-band psychopathology (e.g. internalizing, externalizing; level 2), subscale (e.g. aggression, inattention; level 3).
