# Supplementary material for: A Complex Systems Approach to Causal Discovery in Psychiatry
Source: PLoS One. 2016 Mar 30;11(3):e0151174. doi: 10.1371/journal.pone.0151174 (PMC4814084; doi:10.1371/journal.pone.0151174)
Supplement: S2 File — (DOCX) [file pone.0151174.s002.docx]

**S2 File: Managing Superfluous Information with the CS-CN Method**

Most datasets for psychiatric research contain many variables that measure the same (or close to the same) construct (e.g. several different instruments to measure the same domain of psychopathology). The inclusion of this superfluous information may bias a network analysis because the scaling function of the distribution of the number of links per node is strongly related to the network’s adaptive properties, and the relative importance of a given node to these adaptive properties is related to the number of other nodes that link to it. Accordingly, a set of nodes within a network that measures the same or close to the same construct would be expected to have a disproportionate number of links based on superfluous information. The observed scaling function of the link per node distribution may, therefore, be related to such superfluous information in the dataset. The CS-CN method is programmed to manage this problem as follows:

The aforementioned Variable Table, provided by the investigator prior to the network analysis, contains information about two qualities of each variable that is used to handle this problem of superfluous variables: (1) the construct the variable is thought to measure (e.g. depression from different instruments in the dataset, socioeconomic status measured from parent’s educational level and from parent’s yearly income) and (2) the hierarchical nature of a variable (e.g. an anhedonia subscale of a depression scale, the depression total score of the same scale). Our algorithm disallows variables that measure the same construct to be used in the same network analysis, and also disallows variables in the same hierarchy (e.g. subscale and total score) to be used in the same network analysis. How does it do this? Once the causal network is specified, as described in the methods section, all variables are rank-ordered based on their BC score within the network. Amongst variables that measure the same designated construct, only the variable highest in BC rank is kept, and all others are eliminated. Amongst variables on the same hierarchy, if a lower order variable (e.g. subscale score) emerges with a higher BC rank than its higher order variable (e.g. total score), the higher order variable is eliminated and ALL lower order variables for the given hierarchy are kept. If the higher order variable emerges with a higher BC score than ANY lower order variable in that hierarchy, ALL lower order variables are eliminated and the higher order variable is kept. Importantly, variables thought to measure the same construct at different time points are ALL kept and are designated by time epoch for that construct. Betweenness centrality of a node is defined as the number of shortest paths from all nodes to all other nodes that pass through the given node. It is perhaps the most meaningful index of the importance of a given node to the adaptive functioning of a network.

Why decide to reduce superfluous information at such a late stage rather than to use similar rules for the selection of variables at the beginning? We wanted decisions on variable elimination to be empirically-based rather than to be based on the investigator’s notions of which variables would prove to be more important. The only discretion allowed of the investigator in this process is to categorize variables in the dataset that measure the same or close to the same construct, and the rank of variables within a specific hierarchy, and to indicate this information in the Variable Table. A full description of the Variable Table is offered in S1 File, and a sample Variable Table is offered in S1 Table.
