# Supplementary material for: A Complex Systems Approach to Causal Discovery in Psychiatry
Source: PLoS One. 2016 Mar 30;11(3):e0151174. doi: 10.1371/journal.pone.0151174 (PMC4814084; doi:10.1371/journal.pone.0151174)
Supplement: S3 File — (DOCX) [file pone.0151174.s003.docx]

**S3 File: More Detail on Measuring the Complex Systems Properties of the Causal Network**

The description of Step 2 of the CS-CN method provided in the Methods section describes the properties of CASs we use to assess the causal network produced in Step 1. These properties are outlined in Table 1 of this paper, and indicate whether or not the network has properties consistent with a CAS. Due to space limitations, we were not able to provide details of their measurement in the main body of the text. We use this supplemental section to provide the details, including basic outlines of the relevant algorithms used in Step 2 of the method.

As described in our Methods section, the CS-CN method is programmed to analyze the network properties of the causal network and to compare these properties to their mean values derived from 1000 permutations of a random directed network model. The random directed network model uses the same number of nodes and links as the directed causal network. Our application integrates Matlab BGL [S3-1] to derive each of the network properties described in Table 1. The comparison directed random network models were generated using the modified version of Matlab Tools for Network Analysis [S3-2]. Network visualization is conducted with the Cytoscape platform [S3-3] and cluster analysis and visualization is conducted with the Cytoscape ClusterOne module [S3-4].

Below, we describe each of the metrics used in the analyses presented in this paper:

1. Degree (*k_i_*): the number of links a node (*i*) has to other nodes. In the following equation, N is the total number of nodes in the network, L is the total number of links in the network, and *k_i_* is the degree of node *i*.

1. In-Degree (*k_i_^in^*): the number of links pointing to a node in a directed network.
   1. In-Degree Distribution (*p_k_^in^*): the probability of a randomly selected node having an in-degree value equivalent to a given node’s in-degree (*k_i_^in^*). A graph of this distribution in a CAS will be “scale-free.”
2. Out-Degree (*k_i_^out^*): the number of links coming from a node in a directed network.
   1. Out-Degree Distribution (*p_k_^out^*): the probability of a randomly selected node having an out-degree value equivalent to a given node’s out-degree (*k_i_^out^*). A graph of this distribution in a CAS will be “scale-free.” In the following equations, *N* is the total number of nodes in the network, *p_k_* is the degree distribution relative to node *k,* and *N_k_* is the number of degree *k* nodes.

1. Percent Shortest Path (*d*): the percentage of the network’s paths between any two pairs of nodes that are “shortest paths” (e.g. have the fewest number of links). The shortest path metric is also referred to as distance, geodesic distance, or geodesic path.
2. Characteristic Path Length (*‹d›*): the average shortest distance between all pairs of nodes in the network. In the following equation, *N* is the total number of nodes in the network, *i,j* are the nodes between which path length is being determined, and *d_i,j_* is the shortest path (distance) between nodes *i* and *j*.

1. Network Diameter (*d_max_*): the maximal shortest path in the network (e.g. the largest distance between any pair of nodes).
2. Clustering Coefficient (*C*): the degree to which the neighbors of a given node link to each other. The clustering coefficient of a full network (the average clustering coefficient, represented by *‹C›*) is the average of the clustering coefficients of each node in a network. In the first of the following equations, *L* is the number of links between the *k* neighbors of node *i, k* is the degree of node *i*, *N* is the total number of nodes in the network, and *C_i_* is the clustering coefficient for each node in the network. In the second of the following equations, *i* is the node for which clustering coefficient is being determined, *L* is the number of links between neighbors of node *i*, and *k* is the degree of node *i*.

1. Betweenness Centrality: the number of times any given node must be passed through in order to get from one node to another via shortest paths. .

1. Clusters: highly dense, interconnected, and possibly overlapping regions in a network (e.g. regions of a network where nodes share many edges with one another) [S3-5]. We used the Cytoscape ClusterOne plugin [S3-4] to define clusters. ClusterOne works by identifying regions of a network with high cohesiveness.
2. Random Networks: we used the modified version of Matlab Tools for Network Analysis developed by MIT Strategic Engineering [S3-2] to create permuted (1000 rounds) random networks (e.g. with edges placed between nodes at random) with the same number of nodes and edges as our observed networks. We compared our observed networks to the permuted random networks to demonstrate the Complex Systems properties of our observed networks. Metrics included in the comparison include clustering coefficient, average degree, degree distribution, shortest path distribution, and characteristic path length.

**S3 File References**

1. Gleich D. Matlab BGL v2.1 [software]. Released April 11, 2007. Available from: https://www.cs.purdue.edu/homes/dgleich/packages/matlab_bgl/
2. MIT Strategic Engineering. Matlab Tools for Network Analysis [software]. 2006-2011. Available from: http://strategic.mit.edu/downloads.php?page=matlab_networks.
3. Saito R, Smoot ME, Ono K, Ruscheinski J, Wang PL, Lotia S, et al. (2012) A travel guide to Cytoscape plugins. Nat Methods 9(11): 1069-1076. doi: 10.1038/nmeth.2212.
4. Nepusz T, Yu H, Paccanaro A (2012) Detecting overlapping protein complexes from protein-protein interaction networks. Nat Methods 9(5): 471-472. doi:10.1038/nmeth.1938.
5. Bader GD, Hogue CWV (2003) An automated method for finding molecular complexes in large protein interaction networks*.* BMC Bioinformatics 4: 2. doi: 10.1186/1471-2105-4-2.
