# Supplementary material for: A Complex Systems Approach to Causal Discovery in Psychiatry
Source: PLoS One. 2016 Mar 30;11(3):e0151174. doi: 10.1371/journal.pone.0151174 (PMC4814084; doi:10.1371/journal.pone.0151174)
Supplement: S4 File — (DOCX) [file pone.0151174.s004.docx]

**S4 File: The CHIDS Network Variable Table**

S1 File provided details about the Variable Table to be uploaded with datasets for analysis with the CS-CN method. In S1 Table, we provide a Variable Table for the CHIDS dataset described in our validation study. While this table is not an *exact* replica of the format in which the Variable Table must be uploaded to the web-based platform for conducting the CS-CN analyses, it shows the *type* of information that is required for input. This information includes variable name, time époque, hierarchical structure (total score vs. subscale score on measures), numerical type (continuous vs. categorical), and how the variable was measured at the time of data collection. We coded time époques and hierarchical levels in the following manner:

Time Époque

1 – Constitutional

2 – Pre-Natal

3 – Early Development

4 – Pre-Trauma

5 – Trauma

6 – Acute Hospitalization

7 – Post-Trauma 3 Month Follow-Up

8 – Post-Trauma 12 Month Follow-Up

Hierarchical Structure

1 – Total Score

2 – Broadband Score

3 – Subscale Score

*Note:* If an individual variable is not part of a hierarchically structured set of variables, the default value is 1.

**Descriptions of psychometric measures used to collect CHIDS data**

- Diagnostic Interview for Children and Adolescents (DICA) [S4-1]: a structured diagnostic interview based on the DSM-IV for children ages 6-17.
- Child Behavior Checklist (CBCL) [S4-2]: a 140-item parent-report questionnaire to evaluate behavioral and emotional problems and competencies in children up to age 18.
- Injury Severity Score (ISS) [S4-3]: a brief, well-validated index of injury which is highly correlated to mortality, days spent in hospital, and major surgery.
- Child Dissociative Checklist (CDC) [S4-4]: a 20-item parent/observer-report measure to evaluate dissociative symptoms in children ages 5-12.
- Child Depression Inventory (CDI) [S4-5]: a 28-item self-report interval scale to assess symptoms of depression in children ages 7-17 years.
- Coddington Life Events Scale (LES) [S4-6]: a 36-item self-report measure to assess how specific life events have affected a child’s growth and development.
- Multidimensional Anxiety Scale for Children (MASC) [S4-7]: a 39-item self-report measure addressing pediatric anxiety symptoms.
- FACES Pain Rating Scale [S4-8]: a visual analogue interval scale for children ages 3 and up to indicate level of pain.
- Colored Analogue Pain Scale (CAS) [S4-9]: a visual analogue interval scale for children ages 5 and older to indicate level of pain.
- Peritraumatic Dissociation Scale [S4-10]: an unpublished 18-item interval scale to measure acute peritraumatic dissociation.
- Piers Harris Self-Concept Scale 2 (PH2) [S4-11]: a 60-item self-report interval scale to assess self-concept in children ages 7-18 years.
- UCLA PTSD Reaction Index (PTSDRI) [S4-12]: a structured interview for trauma exposure and DSM-IV PTSD symptoms in children ages 7-17.
- Child Stress Reaction Checklist (CSRC) [S4-13]: a 30-item parent-report measure to assess child traumatic stress.
- Family Strains Scale [S4-14]: a 15-item interval scale to assess stressful changes within a family.
- Brief Symptom Inventory (BSI) [S4-15]: a 53-item self-report measure of a broad range of psychiatric symptoms. Administered to the child’s parents to measure their own level of symptoms.
- PTSD Checklist (PCL) [S4-16]:  is a 17-item self-report measure of the 17 DSM-IV symptoms of PTSD. Administered to the child’s parents to measure their own level of posttraumatic symptoms.
- Stanford Acute Stress Reaction Questionnaire [S4-17]: a 31-item scale to measure acute levels of anxiety and dissociative reactions to traumatic events, following DSM-IV criteria for Acute Stress Disorder. Administered to the child’s parents to measure their own level of acute stress.

**Rule for handling high dimensional genomic data**

If *any* SNP for a given gene is found to be causally related to *any* other variable in the dataset, the gene itself is considered to be causally related to that variable.

**S4 File References**

1. Reich W. Diagnostic interview for children and adolescents (DICA). J Am Acad Child Adolesc Psychiatry. 2000; 39(1): 59-66.
2. Achenbach TM. Manual for the Child Behavior Checklist 4-18 and 1991 Profile. Burlington, VT: University Associates in Psychiatry. 1991.
3. Baker SP, O’Neill B, Haddon W, Long WB. The injury severity score: a method for describing patients with multiple injuries and evaluating emergency care. J Trauma. 1974; 14(3): 189-96.
4. Putnam FW, Peterson G. Further validation of the Child Dissociative Checklist. Dissociation: Progress in the Dissociative Disorders. 1994; 7(4): 204-11.
5. Kovacs M. Children’s Depression Inventory (CDI) Technical Manual. New York, NY: Multi-Health Systems, Inc. 2001.
6. Coddington RD. The significance of life events as etiological factors in the diseases of children: II. A study of a normal population. J Psychosomatic Res. 1972; 16: 205-13.
7. March JS, Parker JD, Sullivan K, Stallings P, Conners CK. The Multidimensional Anxiety Scale for Children (MASC): factor structure, reliability, and validity. J Am Acad Child Adolesc Psychiatry. 1997; 36(4): 554-65.
8. Bieri D, Reeve R, Champion G, Addicoat L, Ziegler J. The Faces Pain Scale for the self-assessment of the severity of pain experienced by children: development, initial validation and preliminary investigation for ration scale properties. Pain. 1990; 41: 139-50.
9. Bulloch B, Garcia-Filion P, Notricia D, Bryson M, McConahay T. Reliability of the Color Analog Scale: Repeatability of Scores in Traumatic and Nontraumatic Injuries. Academic Emergency Medicine. 2009; 16(5): 465-9.
10. Saxe, G. Peritraumatic Dissociation Interval Scale. Unpublished.
11. Piers EV. Piers-Harris 2: Piers Harris Children’s Self-Concept Scale, 2^nd^ Ed. Los Angeles, CA: WPS Publishers. 2002.
12. Pynoos RS, Frederick C, Nader K, Arroyo W, Steinberg A, Eth S, et al. Life Threat and Posttraumatic Stress in School-Age Children. Arch Gen Psychiatry. 1987; 44(12): 1057-63.
13. Saxe G, Chawla N, Stoddard F, Kassam-Adams N, Courtney D, Cunningham K, et al. Child Stress Disorders Checklist: A Measure of ASD and PTSD in Children. J Amer Acad Child Adolesc Psychiatry. 2003; 42(8): 972-8.
14. Moos RHM. Family Environment Scale Manual: Development, applications, research, 3^rd^ Ed. Palo Alto, CA: Consulting Psychologists Press Inc. 1994.
15. Derogatis LR. BSI Brief Symptom Inventory: Administration, Scoring, and Procedure Manual, 4^th^ Ed. Minneapolis, MN: National Computer Systems Pearson, Inc. 1993.
16. Blanchard EB, Jones-Alexander J, Buckley TC, Forneris CA. Psychometric properties of the PTSD checklist (PCL). Behav Res Ther. 1996; 34: 669-73.
17. Cardeña E, Koopman C, Classen C, Waelde LC, Spiegel D. Psychometric properties of the Stanford Acute Stress Reaction Questionnaire (SASRQ): a valid and reliable measure of acute stress. J Trauma Stress. 2000; 13(4): 19-34.
18. Porges SW. Vagal tone: A physiologic marker of stress vulnerability*.* Pediatrics. 1992; 90(3): 498-504.
19. Porges SW. Orienting in a defensive world: Mammalian modifications of our evolutionary heritage. A polyvagal theory*.* Psychophysiology. 1995; 32: 301-18.
20. Miller A, Enlow MB, Reich W, Saxe G. A diagnostic interview for acute stress disorder for children and adolescents. J Trauma Stress. 2009; 22(6): 549-56.
