# Supplementary material for: A Complex Systems Approach to Causal Discovery in Psychiatry
Source: PLoS One. 2016 Mar 30;11(3):e0151174. doi: 10.1371/journal.pone.0151174 (PMC4814084; doi:10.1371/journal.pone.0151174)
Supplement: S5 File — (DOCX) [file pone.0151174.s005.docx]

**S5 File: The Replication Study: NCTSN Core Dataset**

We completed a replication study to determine if the CS-CN method would be applicable to another, much larger dataset related to the development of psychopathology in children. This replication study used the National Child Traumatic Stress Network’s (NCTSN) Core Data Set (CDS).

**Replication Dataset**

The NCTSN CDS contains deidentified data from 14,088 children, aged 0-21, who were seen clinically in one of the participating NCTSN CDS centers. The dataset is maintained by the National Center for Child Traumatic Stress at Duke University Medical Center. The Duke Clinical Research Institute (DCRI) served as the data repository for the NCTSN. The 1,439 variables comprising the portion of the dataset we analyzed describe demographics, residential treatment, family characteristics, service use, trauma exposure, functioning, and standardized assessments of emotional/behavioral problems, trauma related sequalae, and symptoms. Our analysis included variables from 3 follow up visits and was limited only to subjects for whom treatment completion data was available (n=2,093).

**Methods**

We followed the identical 3 Step CS-CN method detailed in the Methods section of the main article.

**Results**

The NCTSN CDS was entered into the CS-CN application and a directed causal network of 962 nodes and 5126 links was produced. This causal network was analyzed for its complex systems properties and compared to the mean value of these properties in a directed random network of 1000 permutations; these network properties are shown in Table A in S5 File.

The data indicate that the properties of this network are not strongly consistent with those of adaptive networks. The observed network does not display small world properties compared to the random network. Its clustering coefficient is, however, more consistent with an adaptive network. This is quite a heterogeneous dataset with a great many variables collected from children from a wide range of different treatment centers around the United States. It may be there is too much heterogeneity in this dataset to discover a complex system without a focus on specific sets of variables. As described in the method section of the primary article, the CS-CN method allows for such a focus. We decided to conduct a focused search starting with the variables describing the children’s exposure to trauma. Since the National Child Traumatic Stress Network’s primary rationale for creating this data was to understand children’s responses to traumatic events, such a focused search was indicated. The NCTSN CDS includes information on trauma exposure from 10 sorts of traumatic events (e.g. sexual abuse, physical abuse, disasters, community violence). We created a subnetwork by searching the initial causal network for nodes that were first neighbors of the 10 trauma variables. This procedure created a smaller network of 224 nodes and 1034 links that we then analyzed for adaptive properties. This causal network was found to have good adaptive properties compared to a model of its random network of 1000 permutations, shown in Figure B in S5 File.

To illustrate its small world properties, we compare its distribution of shortest paths compared to the random network in Figure A in S5 File. About 45% of the shortest paths of the CDS Trauma Causal Network were only 1 or 2 links in length vs. 11% of the shortest paths of the random network. In contrast about 60% of the shortest paths of the random network were 4 links or greater vs. only 25% of the shortest paths of the CDS Trauma Causal Network. We conducted a two-sample Kolmogorov-Smirnov (K.S.) test to determine if these respective distributions were statistically distinct (under the null hypothesis defined by the random network distribution). This analysis confirmed that the distribution of shortest paths of the CDS Trauma Causal Network was very different from that of the random network (K.S.=0.32, p<1X10^-200^).

**Searching for the Network’s Vulnerabilities**

The final step in our approach is to test the adaptive nature of the subnetwork by modeling how it behaves when stressed by the sequential removal of nodes at random, and then to determine the differential response of the network when its most important nodes (defined by highest BC rank) are sequentially removed. The resulting networks, under these two sorts of challenge, are shown in Figure B in S5 File. The CDS Trauma Causal Network maintains its integrity throughout a trial of sequentially removing 30 nodes at random (mean of 1000 random trials). In contrast, when this network is stressed via a trial of node removal by BC rank, it maintains its integrity through the removal of 21 nodes and then its integrity is precipitously lost. The nodes with the highest BC rank were exposure to community violence, neglect, sexual abuse, age at trauma, dissociation, depression, and child’s inappropriate sexual behavior.

**S5 File Figure Legends**

**
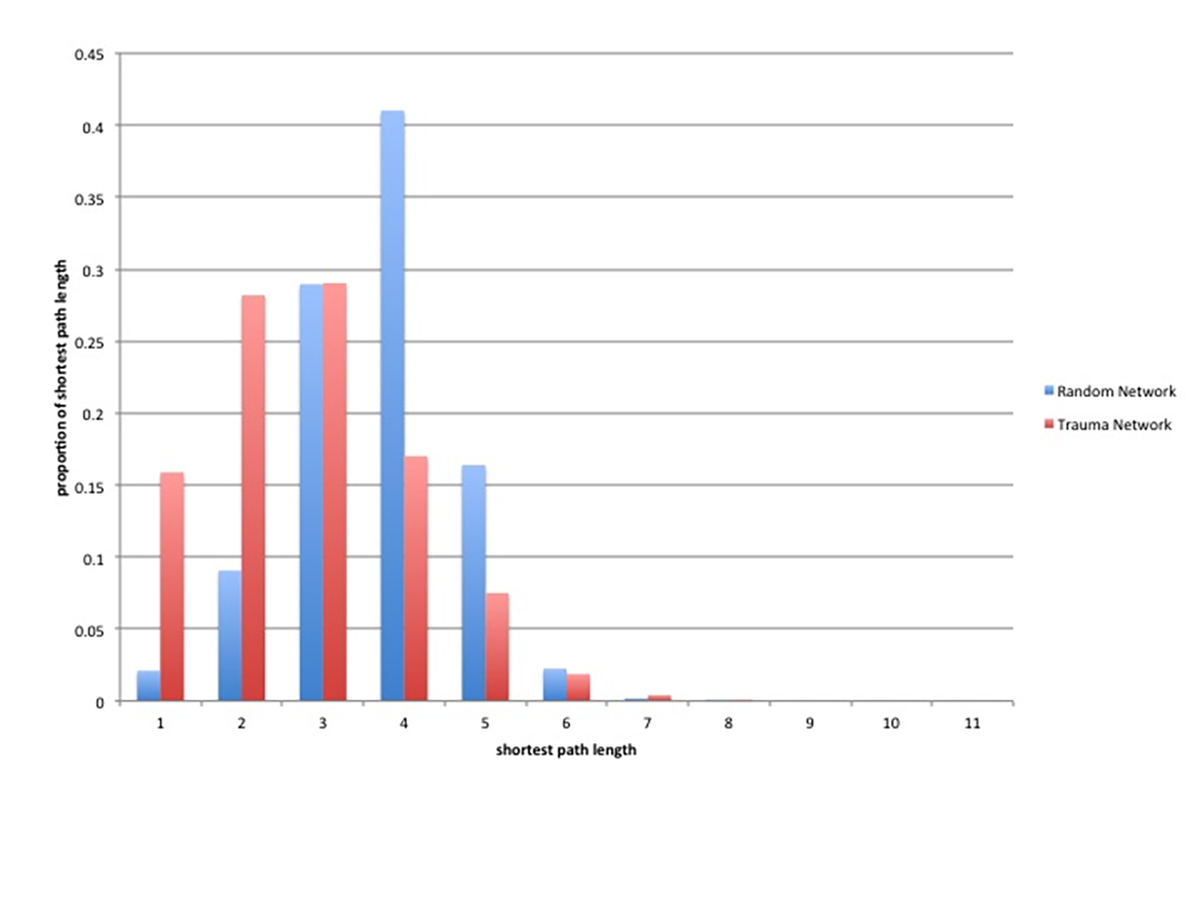
**

**Figure A: Distribution of Shortest Paths in the CDS Trauma Causal Network and the Random Network**

**
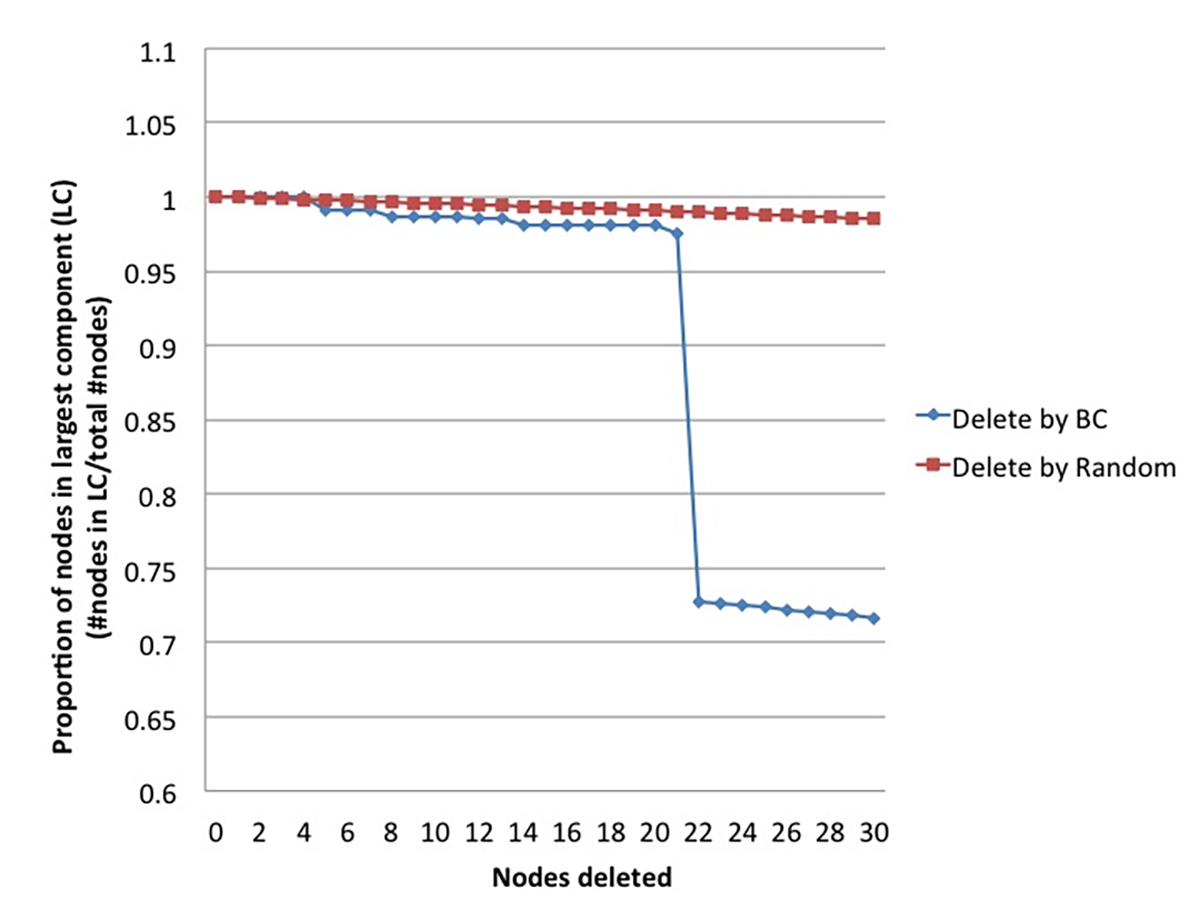
**

**Figure B:** **Integrity of CDS Trauma Causal Network Following Challenge:** The proportion of nodes in the largest network component by sequential removal of 30 nodes at random vs. by BC rank.

**S5 File Tables**

**Table A: Properties of the CDS Causal Network**

| **Network Property** | **CDS Network** | **Random Network** |
| --- | --- | --- |
| Nodes | 962 | 962 |
| Links | 5126 | 5126 |
| Network Diameter | 14 | 9 |
| Characteristic path length | 4.18 | 4.29 |
| Clustering Coefficient | 0.11 | 0.006 |

**Table B: Properties of the CDS Trauma Causal Subnetwork**

| **Network Property** | **CDS Trauma Network** | **Random Network** |
| --- | --- | --- |
| Nodes | 224 | 224 |
| Links | 1034 | 1034 |
| Network Diameter | 8.0 | 7.8 |
| Characteristic path length | 2.8 | 3.7 |
| Clustering Coefficient | 0.15 | 0.02 |
