# Supplementary material for: A Complex Systems Approach to Causal Discovery in Psychiatry
Source: PLoS One. 2016 Mar 30;11(3):e0151174. doi: 10.1371/journal.pone.0151174 (PMC4814084; doi:10.1371/journal.pone.0151174)
Supplement: S6 File — (DOCX) [file pone.0151174.s006.docx]

**S6 File: The Resimulation Controlled Experiment: Validating the CN-CS Method in Comparison to a Causal Discovery “Gold-Standard” Method**

To our knowledge, Causal Discovery algorithms have never before been integrated into a Network Science framework similar to ours. Causal Discovery algorithms have demonstrated remarkable accuracy at identifying the causes of natural phenomena and rest on a solid theoretical foundation not present in commonly applied non-causal analytic frameworks. Will our integration of these algorithms within a Network Science framework sacrifice this accuracy? We conduct a study using a gold standard for causal discovery and compare our CS-CN method hybrid approach to more conventional causal modeling approaches and more conventional statistical approaches for identifying causality within that dataset.

We conducted a controlled experiment on a large, scale-free, realistic biomedical network, REGED, which has been previously used in the international Causality Challenge [S6-1]. Using a network such as REGED with known data-generative causal structure allows us to precisely compute the accuracy of discovery approaches. The REGED network contains 1,000 variables/nodes and 1,148 edges connecting them. Out of 1,000 variables, there are 59 causes of the phenotypic response variable that we seek to identify using various methods. We have generated a dataset with 200 samples from the REGED network and applied the following discovery methods: (1) causal discovery (only) inference by application of local-to-global approach based on global local learning (GLL) algorithm [S6-2]; (2) the CS-CN method defined as causal discovery inference followed by network science analysis (to identify hubs with top 5% connectivity); and established statistical approaches such as (3) ridge regression, (4) elastic net [S6 -3], (5) Lasso [S6 -4], (6) stepwise regression, and (7) graphical Lasso [S6 -5]. The output of each method is compared with the true causes of the phenotypic response variable, and we compute several structural accuracy metrics given in the table below. Details about computation of structural metrics can be found in Bader & Hogue (2003) [S6 -6].

The core 5 metrics, namely, sensitivity, specificity, positive predictive value (PPV), negative predictive value (NPV), and Euclidian distance from the point with perfect sensitivity and PPV are based on computation of true positives (TP), false positives (FP), true negatives (TN), and false negatives (FN). It is desirable to maximize sensitivity, specificity, PPV, NPV and minimize distance.

The results shown in Table A in S6 File imply that causal network induction is dominant over all other approaches for inferring the low-level causal relationships. The integration of Network Science approaches (e.g. the CS-CN method) performs comparably with gold standard causal discovery methods and outperforms established statistical regression-based methods for identification of causes. Furthermore, the CS-CN method provides an additional discovery layer over pure causal discovery because once a high-quality causal structure has been discovered, network analysis then points to higher-level concepts such as robustness and efficiency of information transfer. This result is further corroborated by the analysis of the results of the Validation and Replication Studies described in the main body and S5 File of this manuscript, respectively. Finally, the hybrid CS-CN method has excellent performance, and benefits from distinct but mutually reinforcing aspects of discovery.

**Table A. Comparing Methods for Detecting Known Causes in REGED Network**

|  | **TP** | **FP** | **TN** | **FN** | **Sensitivity** | **Specificity** | **PPV** | **NPV** | **Distance** |
| --- | --- | --- | --- | --- | --- | --- | --- | --- | --- |
| **Causal Discovery method, only** | 29 | 55 | 885 | 30 | 0.49 | 0.94 | 0.35 | 0.97 | 0.58 |
| **CS-CN Method** | 22 | 58 | 882 | 37 | 0.37 | 0.94 | 0.28 | 0.96 | 0.68 |
| **Ridge Regression** | 5 | 78 | 862 | 54 | 0.08 | 0.92 | 0.06 | 0.94 | 0.93 |
| **Elastic Net** | 2 | 9 | 931 | 57 | 0.03 | 0.99 | 0.18 | 0.94 | 0.90 |
| **Lasso** | 2 | 9 | 931 | 57 | 0.03 | 0.99 | 0.18 | 0.94 | 0.90 |
| **Stepwise Regression** | 13 | 176 | 764 | 46 | 0.22 | 0.81 | 0.07 | 0.94 | 0.86 |
| **Graphical Lasso** | 7 | 143 | 797 | 52 | 0.12 | 0.85 | 0.05 | 0.94 | 0.92 |

**S6 File References**

1. Barabasi AL, Martino M, Pósfai M. Graph Theory. In: Barabasi AL, Martino M, Posfai M, editors. Network Science: Interactive Textbook*.* 2012. pp. 21-46. Retrieved from http://barabasilab.neu.edu/networksciencebook/download/network_science_November_Ch2_2012.pdf.
2. Saito R, Smoot ME, Ono K, Ruscheinski J, Wang PL, Lotia S, et al. A travel guide to Cytoscape plugins. Nat Methods. 2012; 9(11): 1069-76.
3. Doncheva NT, Assenov Y, Domingues FS, Albrecht M. Topological analysis and interactive visualization of biological networks and protein structures. Nat Protoc. 2012; 7: 670-85.
4. Borgatti SP, Everett MG. A Graph-theoretic perspective on centrality. Social Networks. 2006; 28: 466-84.
5. Tang Y, Li M, Wang J, Pan Y, Wu F. CytoNCA: a cytoscape plugin for centrality analysis and evaluation of biological networks. BioSystems. 2014; 127: 67-72.
6. Bader GD, Hogue CWV. An automated method for finding molecular complexes in large protein interaction networks. BMC Bioinformatics. 2003; 4: 2.
