# Supplementary material for: A Complex Systems Approach to Causal Discovery in Psychiatry
Source: PLoS One. 2016 Mar 30;11(3):e0151174. doi: 10.1371/journal.pone.0151174 (PMC4814084; doi:10.1371/journal.pone.0151174)
Supplement: S1 Table — (DOCX) [file pone.0151174.s008.docx]

**S1 Table: CHIDS Sample Variable Table**

| **Construct** | **Time Époque** | **Hierarchical Level** | **Variable Type** | **Variable & its Measurement** |
| --- | --- | --- | --- | --- |
| ADRB153 | 1 | 1 | Continuous | ADRB153: SNP rs1801253 on the adrenoceptor beta (ADRB) gene analyzed via buccal DNA samples obtained via mouthwash, isolated using Gentra DNA isolation kit, and typed using real-time PCR technology |
| ADRB178 | 1 | 1 | Continuous | ADRB178: SNP rs2183378 on the adrenoceptor beta (ADRB) gene analyzed via buccal DNA samples obtained via mouthwash, isolated using Gentra DNA isolation kit, and typed using real-time PCR technology |
| AVPR1a15 | 1 | 1 | Continuous | AVPR1a15: SNP rs1042615 on the arginine vasopressin receptor 1a (AVPR1a) gene analyzed via buccal DNA samples obtained via mouthwash, isolated using Gentra DNA isolation kit, and typed using real-time PCR technology |
| AVPR1a20 | 1 | 1 | Continuous | AVPR1a20: SNP rs11174820 on the arginine vasopressin receptor 1a (AVPR1a) gene analyzed via buccal DNA samples obtained via mouthwash, isolated using Gentra DNA isolation kit, and typed using real-time PCR technology |
| AVPR1a39 | 1 | 1 | Continuous | AVPR1a39: SNP rs10784339 on the arginine vasopressin receptor 1a (AVPR1a) gene analyzed via buccal DNA samples obtained via mouthwash, isolated using Gentra DNA isolation kit, and typed using real-time PCR technology |
| AVPR1a46 | 1 | 1 | Continuous | AVPR1a46: SNP rs11836346 on the arginine vasopressin receptor 1a (AVPR1a) gene analyzed via buccal DNA samples obtained via mouthwash, isolated using Gentra DNA isolation kit, and typed using real-time PCR technology |
| AVPR1a50 | 1 | 1 | Continuous | AVPR1a50: SNP rs2738250 on the arginine vasopressin receptor 1a (AVPR1a) gene analyzed via buccal DNA samples obtained via mouthwash, isolated using Gentra DNA isolation kit, and typed using real-time PCR technology |
| AVPR1a54 | 1 | 1 | Continuous | SNP rs2228154 on the arginine vasopressin receptor 1a (AVPR1a) gene analyzed via buccal DNA samples obtained via mouthwash, isolated using Gentra DNA isolation kit, and typed using real-time PCR technology |
| COMT33 | 1 | 1 | Continuous | COMT33: SNP rs4633 on the catechol-O-methyltransferase (COMT) gene analyzed via buccal DNA samples obtained via mouthwash, isolated using Gentra DNA isolation kit, and typed using real-time PCR technology |
| COMT69 | 1 | 1 | Continuous | COMT69: SNP rs6269 on the catechol-O-methyltransferase (COMT) gene analyzed via buccal DNA samples obtained via mouthwash, isolated using Gentra DNA isolation kit, and typed using real-time PCR technology |
| CRHR104 | 1 | 1 | Continuous | CRHR104: SNP rs17763104 on the corticotropin releasing hormone receptor 1 (CRHR1) gene analyzed via buccal DNA samples obtained via mouthwash, isolated using Gentra DNA isolation kit, and typed using real-time PCR technology |
| CRHR112 | 1 | 1 | Continuous | CRHR112: SNP rs12944712 on the corticotropin releasing hormone receptor 1 (CRHR1) gene analyzed via buccal DNA samples obtained via mouthwash, isolated using Gentra DNA isolation kit, and typed using real-time PCR technology |
| CRHR114 | 1 | 1 | Continuous | CRHR114: SNP rs17690314 on the corticotropin releasing hormone receptor 1 (CRHR1) gene analyzed via buccal DNA samples obtained via mouthwash, isolated using Gentra DNA isolation kit, and typed using real-time PCR technology |
| CRHR142 | 1 | 1 | Continuous | CRHR142: SNP rs242942 on the corticotropin releasing hormone receptor 1 (CRHR1) gene analyzed via buccal DNA samples obtained via mouthwash, isolated using Gentra DNA isolation kit, and typed using real-time PCR technology |
| CRHR144 | 1 | 1 | Continuous | CRHR144: SNP rs4458044 on the corticotropin releasing hormone receptor 1 (CRHR1) gene analyzed via buccal DNA samples obtained via mouthwash, isolated using Gentra DNA isolation kit, and typed using real-time PCR technology |
| CRHR158 | 1 | 1 | Continuous | CRHR158: SNP rs17763658 on the corticotropin releasing hormone receptor 1 (CRHR1) gene analyzed via buccal DNA samples obtained via mouthwash, isolated using Gentra DNA isolation kit, and typed using real-time PCR technology |
| CRHR161 | 1 | 1 | Continuous | CRHR161: SNP rs4074461 on the corticotropin releasing hormone receptor 1 (CRHR1) gene analyzed via buccal DNA samples obtained via mouthwash, isolated using Gentra DNA isolation kit, and typed using real-time PCR technology |
| CRHR181 | 1 | 1 | Continuous | CRHR181: SNP rs12936181 on the corticotropin releasing hormone receptor 1 (CRHR1) gene analyzed via buccal DNA samples obtained via mouthwash, isolated using Gentra DNA isolation kit, and typed using real-time PCR technology |
| CRHR192 | 1 | 1 | Continuous | CRHR192: SNP rs11657992 on the corticotropin releasing hormone receptor 1 (CRHR1) gene analyzed via buccal DNA samples obtained via mouthwash, isolated using Gentra DNA isolation kit, and typed using real-time PCR technology |
| FKBP502 | 1 | 1 | Continuous | FKBP502: SNP rs4713902 on the FK506 binding protein 5 (FKBP5) gene analyzed via buccal DNA samples obtained via mouthwash, isolated using Gentra DNA isolation kit, and typed using real-time PCR technology |
| FKBP524 | 1 | 1 | Continuous | FKBP524:SNP rs9380524 on the FK506 binding protein 5 (FKBP5) gene analyzed via buccal DNA samples obtained via mouthwash, isolated using Gentra DNA isolation kit, and typed using real-time PCR technology |
| FKBP533 | 1 | 1 | Continuous | FKBP533: SNP rs6926133 on the FK506 binding protein 5 (FKBP5) gene analyzed via buccal DNA samples obtained via mouthwash, isolated using Gentra DNA isolation kit, and typed using real-time PCR technology |
| FKBP534 | 1 | 1 | Continuous | FKBP534: SNP rs10498734 on the FK506 binding protein 5 (FKBP5) gene analyzed via buccal DNA samples obtained via mouthwash, isolated using Gentra DNA isolation kit, and typed using real-time PCR technology |
| FKBP542 | 1 | 1 | Continuous | FKBP542: SNP rs17614642 on the FK506 binding protein 5 (FKBP5) gene analyzed via buccal DNA samples obtained via mouthwash, isolated using Gentra DNA isolation kit, and typed using real-time PCR technology |
| FKBP547 | 1 | 1 | Continuous | FKBP547: SNP rs3777747 on the FK506 binding protein 5 (FKBP5) gene analyzed via buccal DNA samples obtained via mouthwash, isolated using Gentra DNA isolation kit, and typed using real-time PCR technology |
| FKBP558 | 1 | 1 | Continuous | FKBP558: SNP rs9296158 on the FK506 binding protein 5 (FKBP5) gene analyzed via buccal DNA samples obtained via mouthwash, isolated using Gentra DNA isolation kit, and typed using real-time PCR technology |
| FKBP563 | 1 | 1 | Continuous | FKBP563: SNP rs10947563 on the FK506 binding protein 5 (FKBP5) gene analyzed via buccal DNA samples obtained via mouthwash, isolated using Gentra DNA isolation kit, and typed using real-time PCR technology |
| FKBP573 | 1 | 1 | Continuous | FKBP573: SNP rs3800373 on the FK506 binding protein 5 (FKBP5) gene analyzed via buccal DNA samples obtained via mouthwash, isolated using Gentra DNA isolation kit, and typed using real-time PCR technology |
| OPRM171 | 1 | 1 | Continuous | OPRM171: SNP rs1799971 on the opioid receptor, mu1 (OPMR1) gene analyzed via buccal DNA samples obtained via mouthwash, isolated using Gentra DNA isolation kit, and typed using real-time PCR technology |
| Preg Alc | 2 | 1 | Continuous | How frequently mother drank anything with alcohol during pregnancy as recorded on the 49-item Pregnancy section of the Diagnostic Interview for Children and Adolescents (**DICA**) |
| Preg Drinks | 2 | 1 | Continuous | Mother’s maximum number of alcoholic drinks at one time during pregnancy as recorded on the 49-item Pregnancy section of the **DICA** |
| Preg Ciggs | 2 | 1 | Continuous | Number of cigarettes mother smoked during pregnancy as recorded on the 49-item Pregnancy section of the **DICA** |
| Emo Preg Probs | 2 | 1 | Categorical | Emotional problems (depression, anxiety) during pregnancy for which mother sought treatment or counseling as recorded on the 49-item Pregnancy section of the **DICA** |
| Preg Probs | 2 | 1 | Continuous | Mother’s total number of problems during pregnancy as recorded on the 49-item Pregnancy section of the **DICA** |
| Gender | 1 | 1 | Categorical | Child’s gender |
| Birth Weight | 3 | 1 | Continuous | Child’s birth weight in pounds and ounces as recorded on the 8-item Birth section of the **DICA** |
| Premature | 3 | 1 | Categorical | Birth of child was premature as recorded on the 8-item Birth section of the **DICA** |
| Happy Baby | 3 | 1 | Categorical | How happy child was as a baby/whether child gave few problems as recorded on the 12-item Infancy section of the **DICA** |
| Difficult Baby | 3 | 1 | Categorical | Child was difficult during 1^st^ year as recorded on the 12-item Infancy section of the **DICA** |
| Breast Feeding | 3 | 1 | Categorical | Whether child was breastfed as recorded on the 12-item Infancy section of the **DICA** |
| Breast Feeding Wks | 3 | 1 | Continuous | Number of weeks child was breastfed as recorded on the 12-item Infancy section of the **DICA** |
| Crawl | 3 | 1 | Continuous | Age in months at which child first crawled as recorded on the 12-item Early Development section of the **DICA** |
| Sit Up | 3 | 1 | Continuous | Age in months at which child first sat up as a baby as recorded on the 12-item Early Development section of the **DICA** |
| Walk | 3 | 1 | Continuous | Age in months at which child first walked as recorded on the 12-item Early Development section of the **DICA** |
| Crying | 3 | 1 | Continuous | How much child cried (was never satisfied) as a baby as recorded on the 12-item Infancy section of the **DICA** |
| Sleep | 3 | 1 | Categorical | Parent had trouble getting child to sleep at night as recorded on the 12-item Sleep Disorders section of the **DICA** |
| Panic | 3 | 1 | Categorical | Child was troubled by panic states in the middle of the night as recorded on the 12-item Sleep Disorders section of the **DICA** |
| Words | 3 | 1 | Continuous | Age in months at which child first used understandable words as recorded on the 12-item Early Development section of the **DICA** |
| Sentences | 3 | 1 | Continuous | Age in months at which child first used complete sentences as recorded on the 12-item Early Development section of the **DICA** |
| Kindergarten | 3 | 1 | Categorical | Child attended kindergarten as recorded on the 16-item Year-One-Through-Six section of the **DICA** |
| Dev Probs | 3 | 1 | Continuous | Total number of child problems as recorded on the 16-item Year-One-Through-Six section of the **DICA** |
| Held Back | 4 | 1 | Categorical | Child has been held back in school as recorded on the 31-item Demographics section of the **DICA** |
| IEP | 4 | 1 | Categorical | Child has had or has Individual Education Plan as recorded on the 31-item Demographics section of the **DICA** |
| Sports | 4 | 1 | Categorical | Child plays a sport as recorded on the 22-item demographics section of the **DICA** |
| Worship | 4 | 1 | Ordinal Scale | Child attends church, synagogue, or other place of worship as recorded on the 22-item demographics section of the **DICA** |
| Sick | 4 | 1 | Categorical | Child ever sick/ injured or had to go to hospital or stay home a long time as recorded on the 31-item Demographics section of the **DICA** |
| PTSD Hist | 4 | 1 | Continuous | Child’s past PTSD symptoms, as recorded on the 24-item PTSD section of the **DICA** |
| Counseling | 4 | 1 | Categorical | Child talked to someone (counselor, doctor, rabbi, priest, etc.) about troubles as recorded on the 22-item demographics section of the **DICA** |
| SES | 4 | 1 | Continuous | Socioeconomic status of child’s family as recorded on the Child Behavior Checklist (**CBCL**) |
| People in Hm | 4 | 1 | Continuous | Number of people that live in child’s home as recorded on the 31-item Demographics section of the **DICA** |
| Age at Trauma | 5 | 1 | Continuous | Age of child in years at time of trauma as recorded on child’s medical record |
| Head Inj | 5 | 1 | Categorical | Child suffered head Injury as recorded on child’s medical record |
| Violent Inj | 5 | 1 | Categorical | Child suffered violent injury (stabbing, assault, gunshot wound) or non-violent injury (all others) as recorded on child’s medical record |
| Inj Severity | 5 | 1 | Continuous | Child’s Injury Severity Score as recorded by child’s admitting surgeon on child’s medical record |
| Hosp Stay | 6 | 1 | Continuous | Length of stay in hospital in days following injury as recorded on child’s medical record |
| Heart Rate | 6 | 1 | Continuous | Average heart rate over hospital stay as recorded by child’s nurse on child’s medical record |
| Diastolic BP | 6 | 1 | Continuous | Average diastolic blood pressure over hospital stay as recorded by child’s nurse on child’s medical record |
| Systolic BP | 6 | 1 | Continuous | Average systolic blood pressure over hospital stay as recorded by child’s nurse on child’s medical record |
| Pulse | 6 | 1 | Continuous | Pulse during entire length of hospital stay as recorded on child’s medical record |
| 8am Cortisol | 6 | 1 | Continuous | Blood sample collected via Qaigen Kits and shipped to Children’s Hospital Boston (CHB) Molecular Genetics Core Facility for cortisol analysis |
| Cortisol | 6 | 1 | Continuous | Saliva sample collected via Oragene Kit and shipped to CHB Molecular Genetics Core Facility for cortisol analysis |
| Benzos | 6 | 1 | Continuous | Benzodiazepine use (mg/kg/total) during total length of hospital stay as recorded on child’s medical record |
| Ketamine | 6 | 1 | Continuous | Ketamine use (mg/kg/total) during total length of hospital stay as recorded on child’s medical record |
| Morphine | 6 | 1 | Continuous | Morphine use (mg/kg/total) during total length of hospital stay as recorded on child’s medical record |
| Morphine Self | 6 | 1 | Categorical | Whether child self-administered morphine during total length of hospital stay as recorded on child’s medical record |
| Heart Var-Base | 6 | 1 | Continuous | Heart beat-to-beat variability at baseline stress test. Measured by Minimeter physiologic monitor. Stress procedure involved exposing child to 3 sequential 1 minute video clips. The first video clip was a neutral pleasant clip. The second exposed the child to a clip reminiscent of their injury. The third repeated the neutral clip. Heart Var-Base was the measure of heart beat-to-beat variability during the neutral video clip. [S4-18-19] |
| Heart Var-Stress | 6 | 1 | Continuous | Heart Var-Stress was the measure of heart beat-to-beat variability during the 1 minute video clip reminiscent of the child’s injury, in the Stress Procedure defined above. [S4-18-19] |
| Heart Var-Recov | 6 | 1 | Continuous | Heart Var-Recovery was the measure of heart beat-to-beat variability during the presentation of the 1minute neural video clip that followed the 1minute stress video clip, in the Stress Procedure defined above. [S4-18-19] |
| Mean MHPG Values | 6 | 1 | Continuous | Mean MHPG levels collected via Oragene kit and shipped to CHB Molecular Genetics Core Facility for cortisol analysis |
| ASD Diagnosis | 6 | 1 | Categorical | Whether child was diagnosed with Acute Stress Disorder (ASD) from the ASD Module of the DICA [S4-20] |
| Act Comp Ac | 6 | 4 | Continuous | Child’s score on the Activities Competence Scale of the **CBCL** during acute hospitalization following injury |
| Act Comp 3 | 7 | 4 | Continuous | Child’s score on the Activities Competence Scale of the **CBCL** 3 months after injury |
| Act Comp 12 | 8 | 4 | Continuous | Child’s score on the Activities Competence Scale of the **CBCL** 12 months after injury |
| Sch Comp Ac | 6 | 4 | Continuous | Child’s score on the School Competence Scale of the **CBCL** during acute hospitalization following injury |
| Sch Comp 3 | 7 | 4 | Continuous | Child’s score on the School Competence Scale of the **CBCL** 3 months after injury |
| Sch Comp 12 | 8 | 4 | Continuous | Child’s score on the School Competence Scale of the **CBCL** 12 months after injury |
| Soc Comp Ac | 6 | 4 | Continuous | Child’s score on the Social Competence Scale of the **CBCL** during acute hospitalization following injury |
| Soc Comp 3 | 7 | 4 | Continuous | Child’s score on the Social Competence Scale of the **CBCL** 3 months after injury |
| Soc Comp 12 | 8 | 4 | Continuous | Child’s score on the Social Competence Scale of the **CBCL** 12 months after injury |
| PTSD Ac | 6 | 3 | Continuous | Child’s score on the PTSD Scale of the **CBCL** during acute hospitalization following injury |
| PTSD 3 | 7 | 3 | Continuous | Child’s score on the PTSD Scale of the **CBCL** 3 months after injury |
| PTSD 12 | 8 | 3 | Continuous | Child’s score on the PTSD Scale of the **CBCL** 12 months after injury |
| Externalizing Ac | 6 | 2 | Continuous | Child’s Externalizing score on the **CBCL** during acute hospitalization following injury |
| Externalizing 3 | 7 | 2 | Continuous | Child’s Externalizing score on the **CBCL** 3 months after injury |
| Externalizing 12 | 8 | 2 | Continuous | Child’s Externalizing score on the **CBCL** 12 months after injury |
| Internalizing Ac | 6 | 2 | Continuous | Child’s Internalizing score on the **CBCL** during acute hospitalization following injury |
| Internalizing 3 | 7 | 2 | Continuous | Child’s Internalizing score on the **CBCL** 3 months after injury |
| Internalizing 12 | 8 | 2 | Continuous | Child’s Internalizing score on the **CBCL** 12 months after injury |
| Tot Comp 12 | 8 | 1 | Continuous | Child’s total score on the **CBCL** 12 months after injury |
| Dissociation Ac | 6 | 3 | Continuous | Child’s score on the Child Dissociative Checklist (**CDC**) during acute hospitalization following injury |
| Dissociation 3 | 7 | 3 | Continuous | Child’s score on the **CDC** 3 months after injury |
| Dissociation 12 | 8 | 3 | Continuous | Child’s score on the **CDC** 12 months after injury |
| Depression Ac | 6 | 1 | Continuous | Child’s total score on the Child Depression Inventory (**CDI**) during acute hospitalization following injury |
| Depression 3 | 7 | 1 | Continuous | Child’s total score on the **CDI** 3 months after injury |
| Depression 12 | 8 | 1 | Continuous | Child’s total score on the **CDI** 12 months after injury |
| Diagnosis 3 | 7 | 1 | Continuous | Child’s diagnosis as recorded on the **DICA** 3 months after injury |
| Diagnosis 12 | 8 | 1 | Continuous | Child’s diagnosis as recorded on the **DICA** 12 months after injury |
| Death | 4 | 3 | Continuous | Child’s sum of death questions on the Coddington Life Events Interval Scale (**LES**) |
| Family Stress | 4 | 3 | Continuous | Child’s sum of family stress questions on the **LES** |
| Sch Func | 4 | 3 | Continuous | Child’s sum of school questions on the **LES** |
| Life Events Top 10 | 4 | 2 | Continuous | Child’s sum of top 10 highest scored questions on the **LES** |
| Life Events Tot | 4 | 1 | Continuous | Child’s total score on the **LES** |
| Harm Avoidance Ac | 6 | 3 | Continuous | Child’s score on the Harm Avoidance Scale of the Multidimensional Anxiety Interval Scale for Children (**MASC**) during acute hospitalization following injury |
| Harm Avoidance 3 | 7 | 3 | Continuous | Child’s score on the Harm Avoidance Scale of the **MASC** 3 months after injury |
| Harm Avoidance 12 | 8 | 3 | Continuous | Child’s score on the Harm Avoidance Scale of the **MASC** 12 months after injury |
| Physical Symptoms 12 | 8 (Post-Trauma 12 Months) | 3 | Continuous | Child’s score on the Physical Symptoms Scale of the **MASC** 12 months after injury |
| Social Anxiety Ac | 6 | 3 | Continuous | Child’s score on the Social Anxiety Scale of the **MASC** during acute hospitalization following injury |
| Social Anxiety 3 | 7 | 3 | Continuous | Child’s score on the Social Anxiety Scale of the **MASC** 3 months after injury |
| Social Anxiety 12 | 8 | 3 | Continuous | Child’s score on the Social Anxiety Scale of the **MASC** 12 months after injury |
| Separation Anxiety Ac | 6 | 3 | Continuous | Child’s score on the Separation Anxiety Scale of the **MASC** during acute hospitalization following injury |
| Separation Anxiety 3 | 7 | 3 | Continuous | Child’s score on the Separation Anxiety Scale of the **MASC** 3 months after injury |
| Separation Anxiety 12 | 8 | 3 | Continuous | Child’s score on the Separation Anxiety Scale of the **MASC** 12 months after injury |
| Anxiety Tot | 6 | 1 | Continuous | Child’s total score on the **MASC** during acute hospitalization following injury |
| Anxiety Tot 3 | 7 | 1 | Continuous | Child’s total score on the **MASC** 3 months after injury |
| Anxiety Tot 12 | 8 | 1 | Continuous | Child’s total score on the **MASC** 12 months after injury |
| Pain Ac | 6 | 1 | Continuous | Child’s score on the FACES Pain Rating Scale (**FACES**) during acute hospitalization following injury |
| Pain 3 | 7 | 1 | Continuous | Child’s score on the **FACES** 3 months after injury |
| Pain 12 | 8 | 1 | Continuous | Child’s score on the **FACES** 12 months after injury |
| Pain Ac | 6 | 1 | Continuous | Child’s Pain Visual Analogue Interval Scale score collected by interactive measure (Colored Analogue Pain Interval Scale (**CAS**)) during acute hospitalization following injury |
| Pain 3 | 7 | 1 | Continuous | Child’s pain score collected by the **CAS** 3 months after injury |
| Pain 12 | 8 | 1 | Continuous | Child’s pain score collected by the **CAS** 12 months after injury |
| Peritr Disso | 5 | 1 | Continuous | Total score on the Peritraumatic Dissociation Interval Scale |
| Phys App Ac | 6 | 3 | Continuous | Child’s score on the Physical Appearance and Attributes Interval Scale of the Piers-Harris Children’s Self-Concept Interval Scale, 2^nd^ Ed. (**PH2**) during acute hospitalization following injury |
| Phys App 3 | 7 | 3 | Continuous | Child’s score on the Physical Appearance and Attributes Interval Scale of the **PH2** 3 months after injury |
| Phys App 12 | 8 | 3 | Continuous | Child’s score on the Physical Appearance and Attributes Interval Scale of the **PH2** 12 months after injury |
| Hap & Sat Ac | 6 | 3 | Continuous | Child’s score on the Happiness and Satisfaction Interval Scale of the **PH2** during acute hospitalization following injury |
| Hap & Sat 3 | 7 | 3 | Continuous | Child’s score on the Happiness and Satisfaction Interval Scale of the **PH2** 3 months after injury |
| Hap & Sat 12 | 8 | 3 | Continuous | Child’s score on the Happiness and Satisfaction Interval Scale of the **PH2** 12 months after injury |
| Self-Concept Ac | 6 | 1 | Continuous | Child’s total score on the **PH2** during acute hospitalization following injury |
| Self-Concept 3 | 7 | 1 | Continuous | Child’s total score on the **PH2** 3 months after injury |
| Self-Concept 12 | 8 | 1 | Continuous | Total score on the **PH2** 12 months after injury |
| PTSD Ac | 6 | 3 | Continuous | Child’s score on the first 20 items of the PTSD Reaction Index (**PTSDRI**) during acute hospitalization following injury |
| PTSD 3 | 7 | 3 | Continuous | Child’s score on the first 20 items of the **PTSDRI** 3 months after injury |
| PTSD 12 | 8 | 3 | Continuous | Child’s score on the first 20 items of the **PTSDRI** 12 months after injury |
| PTSD Ac | 6 | 1 | Cotinuous | Child’s PTSD symptom score on the Child Stress Reaction Checklist (**CSRC**), Parent Report, during acute hospitalization following injury |
| PTSD 3 | 7 | 1 | Continuous | Child’s PTSD symptom score on the **CSRC**, Parent Report, 3 months after injury |
| PTSD 12 | 8 | 1 | Continuous | Child’s PTSD symptom score on the **CSRC**, Parent Report, 12 months after injury |
| Family Stress Ac | 6 | 1 | Continuous | Family score on the **Family Strains Scale** during acute hospitalization following injury |
| Family Stress 3 | 7 (Post-Trauma 3 Months) | 1 | Continuous | Family score on the **Family Strains Scale** 3 months after injury |
| Family Stress 12 | 8 (Post-Trauma 12 Months) | 1 | Continuous | Family score on the **Family Strains Scale** 12 months after injury |
| P Dep Ac | 6 | 3 | Continuous | Parent’s own depression score on the Brief Symptom Inventory (**BSI**) during child’s acute hospitalization following injury |
| P Dep 3 | 7 | 3 | Continuous | Parent’s own depression score on the **BSI** 3 months after child’s injury |
| P Dep 12 | 8 | 3 | Continuous | Parent’s own depression Score on the **BSI** 12 months after child’s injury |
| P Pos Sym Ac | 6 | 3 | Continuous | Parent’s own positive symptoms score on the **BSI** during acute hospitalization following child’s injury |
| P Pos Symp 3 | 7 | 3 | Continuous | Parent’s own positive symptoms score on the **BSI** 3 months after child’s injury |
| P Pos Symp 12 | 8 | 3 | Continuous | Parent’s own positive symptoms score on the **BSI** 12 months after child’s injury |
| P Global Sev Ac | 6 | 2 | Continuous | Parent’s own Global Severity Index T-score on the **BSI** during acute hospitalization following child’s injury |
| P Global Sev 3 | 7 | 2 | Continuous | Parent’s own Global Severity Index T-score on the **BSI** 3 months after child’s injury |
| P Global Sev 12 | 8 | 2 | Continuous | Parent’s own Global Severity Index T-score on the **BSI** 12 months after child’s injury |
| P PTSD 3 | 7 | 1 | Continuous | Parent’s own score on the PTSD Checklist (**PCL**) 3 months after child’s injury |
| P PTSD 12 | 8 | 1 | Continuous | Parent’s own score on the **PCL** 12 months after injury |
| P Acute Str | 6 | 1 |  | Parent’s own score on the **Stanford Acute Stress Reaction Questionnaire (SASRQ)** during acute hospitalization following child’s injury |
